# Supplementary material for: Weighted likelihood inference of genomic autozygosity patterns in dense genotype data
Source: BMC Genomics. 2017 Dec 1;18:928. doi: 10.1186/s12864-017-4312-3 (PMC5709839; doi:10.1186/s12864-017-4312-3)
Supplement: Supplementary file 1 — Supplementary figures and their legends. (PDF 38834 kb) [file 12864_2017_4312_MOESM1_ESM.pdf]

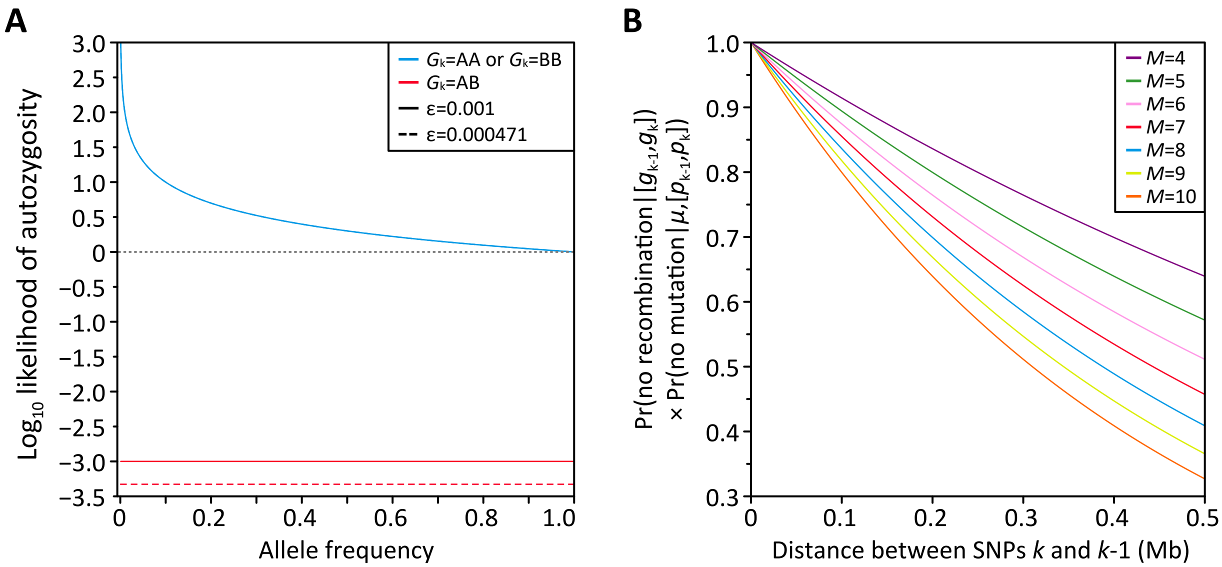

Figure S1. Properties of the likelihood-based estimators. (**A**) The effect of allele frequency on the log-likelihood of autozygosity (equation 1) for homozygous and heterozygous genotypes (**Table 1**) with ε equal to 0.001 [18] and 4.71×10^{-4} (this study). Note that the line for homozygous genotypes with $\varepsilon=4.71\times10^{-4}$ overlaps the line with $\varepsilon=0.001$. (**B**) The magnitude of the product of the probabilities of recombination (equation 3) and mutation (equation 4) as a function of the distance separating a pair of SNPs, assuming a fixed crossover rate of one centimorgan (cM) per one million base pairs and a sex-averaged mutation rate of 1.18×10^{-8} [162].

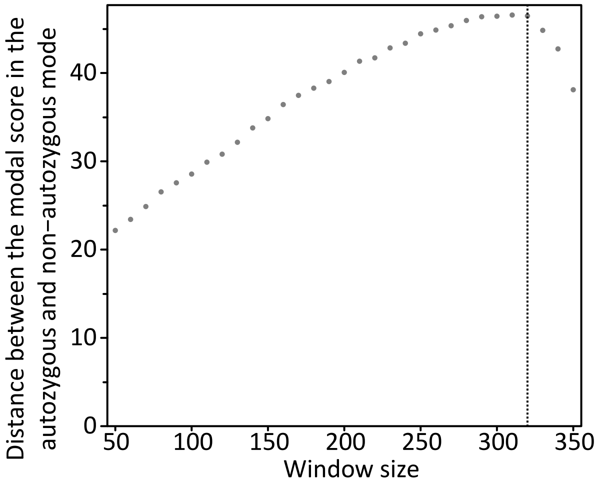

Figure S2. Intermodal distances in the Puerto Rican population. The change in intermodal distance with increasing window size in the PUR population is shown for the Omni2.5 dataset. The vertical dotted line indicates the window size at which the maximum intermodal distance was attained (320 SNPs).

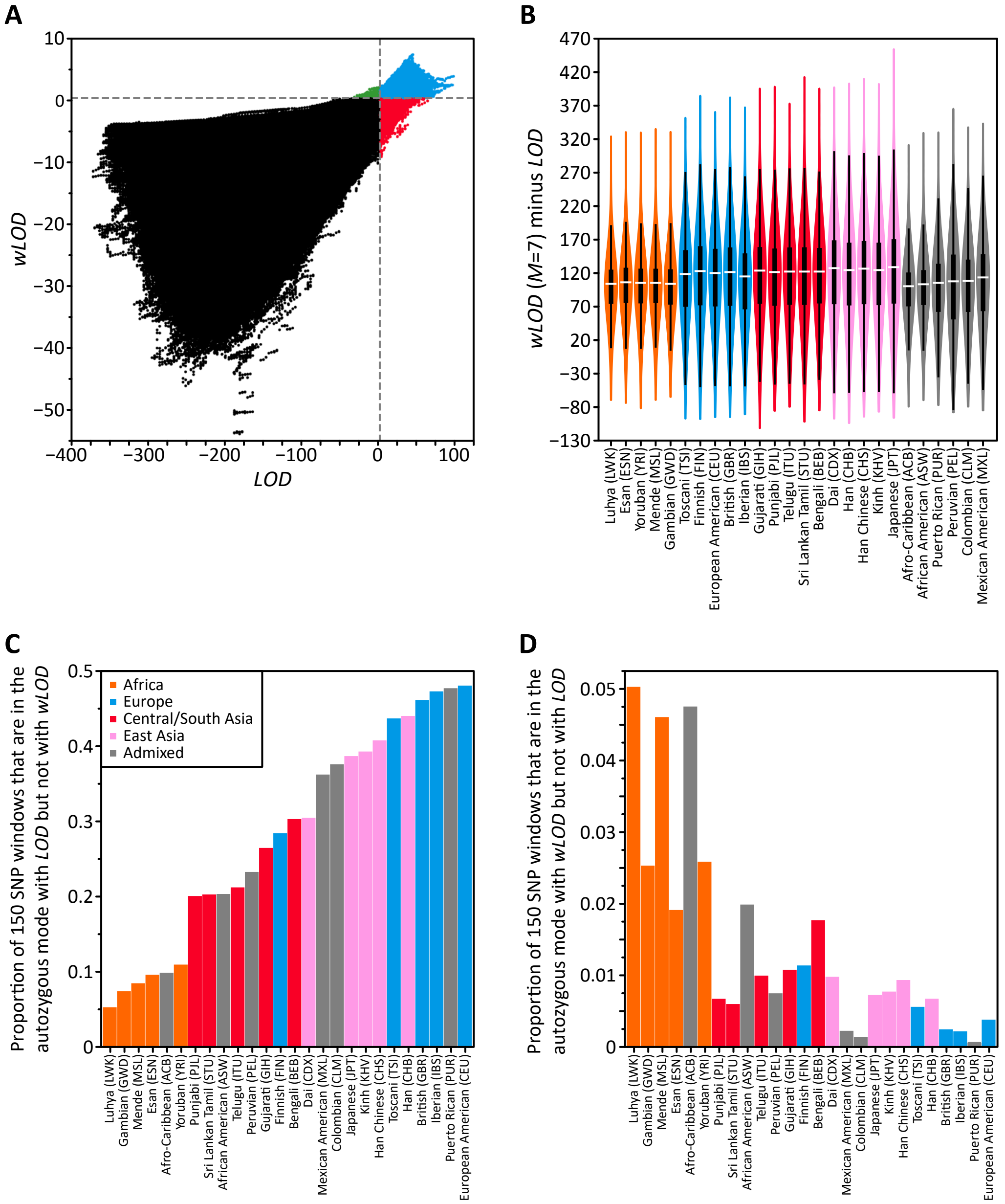

Figure S3. Difference in per-window scores between the LOD and $wLOD$ estimators. (**A**) Scatterplot comparing per-window LOD and $wLOD$ scores across all individuals in the European American (CEU) population at a window size of 150 SNPs in the Omni2.5 dataset. The 2,675,059 windows that were in the autozygous mode with both LOD and $wLOD$ are shown in blue. The 9,885 windows that were in the non-autozygous mode with LOD but the autozygous mode with $wLOD$ are shown in green. The 2,462,843 windows that were in the autozygous mode with LOD but the non-autozygous mode with $wLOD$ are shown in red. All windows that were in the non-autozygous mode with both LOD and $wLOD$ are shown in black. (**B**) Violin plots representing the change in per-window score between $wLOD$ and LOD across all individuals in each population for 150 SNP windows in the Omni2.5 dataset. Each ''violin'' contains a vertical black line (25%–75% range) and a horizontal white line (median), with the width depicting a 90º-rotated kernel density trace and its reflection, both colored by the geographic affiliation of the population [252]. Bar plots showing for each population the proportion of 150 SNP windows that are (**C**) in the autozygous mode with the LOD estimator but are in the non-autozygous mode with the $wLOD$ estimator or (**D**) in the non-autozygous mode with the LOD estimator but are in the autozygous mode with the $wLOD$ estimator.

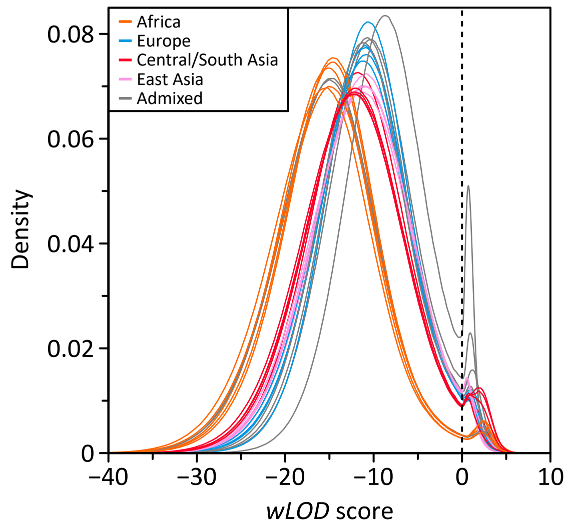

Figure S4. $wLOD$ score distributions in 26 Populations. For the largest window size that had a bimodal distribution of $wLOD$ scores in all 26 populations included in The 1000 Genomes Project Phase 3, separate line graphs representing Gaussian kernel density estimates of the pooled $wLOD$ scores from all individuals in a given population are shown, colored by its geographic affiliation.

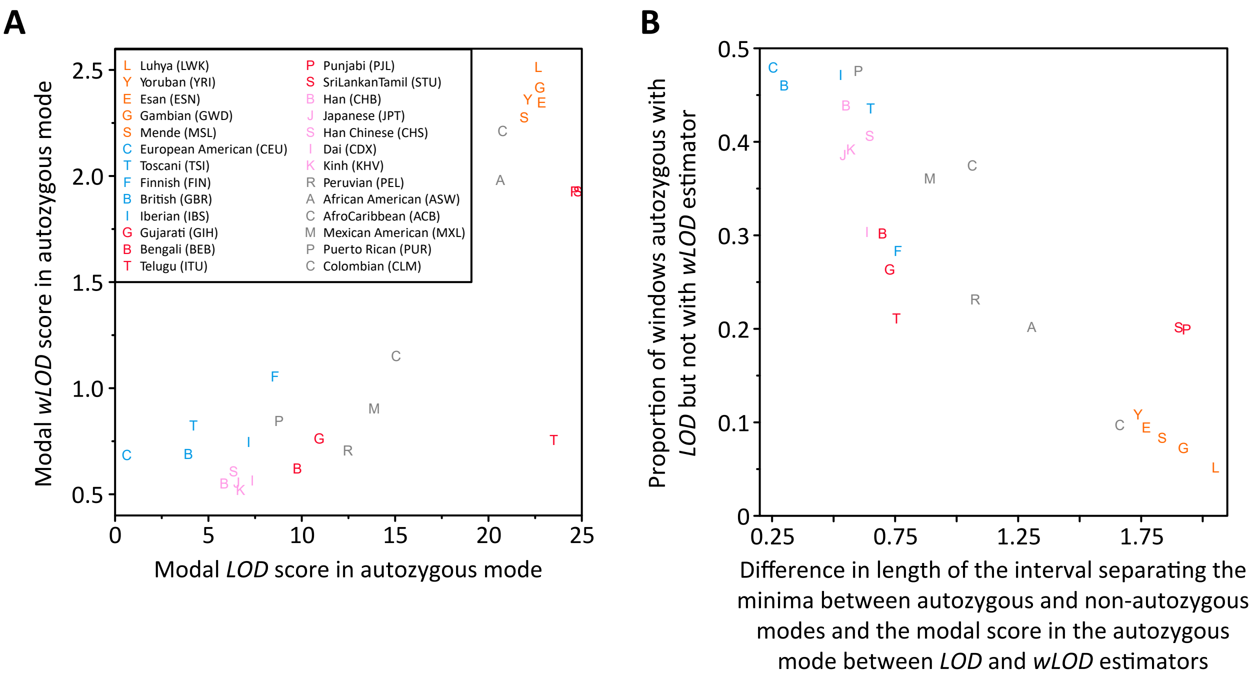

Figure S5. Difference in modal score in the autozygous mode with the LOD and $wLOD$ estimators. Scatterplots comparing (**A**) the modal score in the autozygous mode with the LOD and $wLOD$ estimators and (**B**) the proportion of windows that transition from the autozygous to the non-autozygous mode with the $wLOD$ estimator against the difference in distance between the location of the inter-modal minimum and the modal score in the autozygous mode with the LOD and $wLOD$ estimators for the Omni2.5 dataset. Each population is represented as a single point colored by its geographic region affiliation: Africa, orange; Europe, blue; Central/South Asia, red; East Asia, pink; admixed, grey.

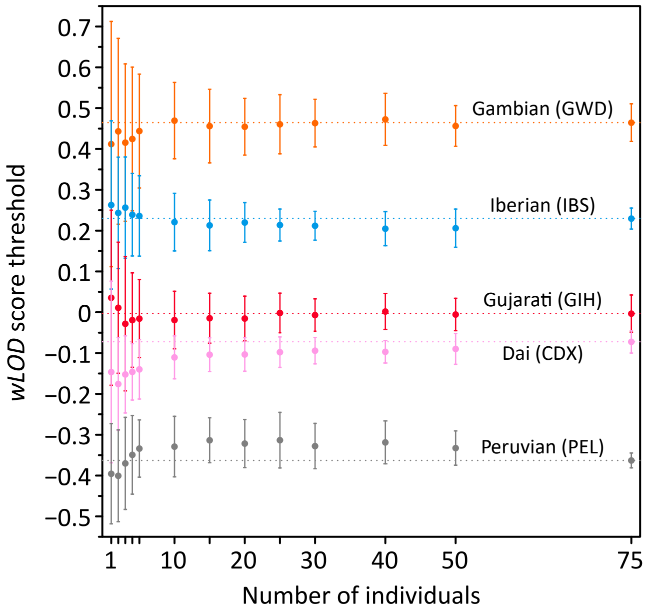

Figure S6. Effect of sample size on $wLOD$ score threshold. Scatterplots depicting the effect of sample sizes on the location of the intermodal minimum between the non-autozygous and autozygous mode in the $wLOD$ score distribution for the Omni2.5 dataset. A representative population from each geographic region and among the six admixed populations is shown. At each sample size 100 random samples of individuals were analyzed. Each point represents the mean across the 100 replicates at that sample size and the vertical line indicates the interval bounded by mean ± 1 SD. Points and lines are colored by the geographic affiliation of the population: Africa, orange; Europe, blue; Central/South Asia, red; East Asia, pink; admixed, grey.

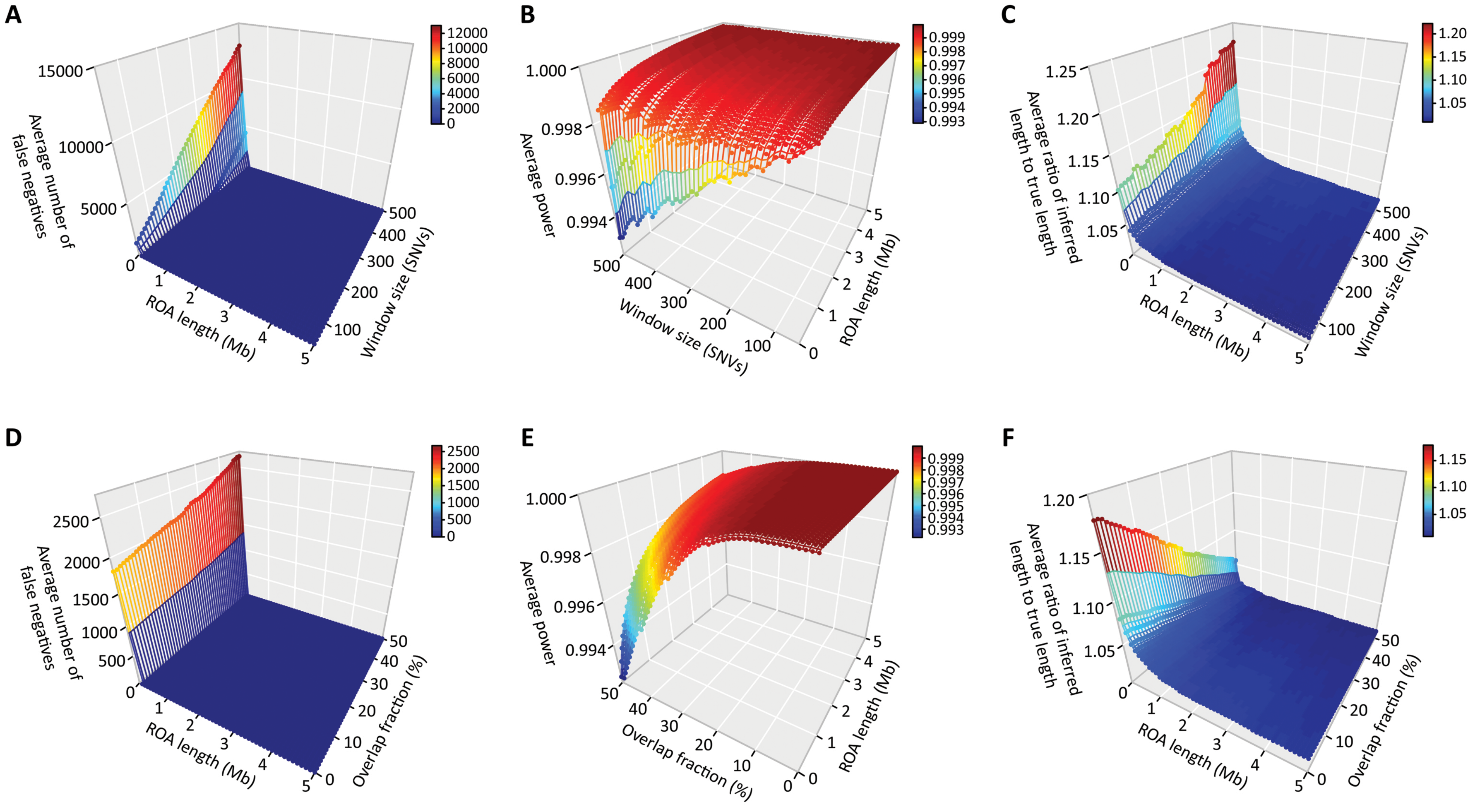

Figure S7. Effect of increasing window size and overlap fraction on detection of ROA of different lengths. For scenario 1 and simulated datasets containing ~750,000 SNVs, 3D scatterplots depicting how window size (**A-C**) and overlap fraction (**D-F**) affect the average number of false negative calls (**A & B**), power (**B & E**), and ratio of inferred to true length (**C & F**) for ROA of different lengths are shown.

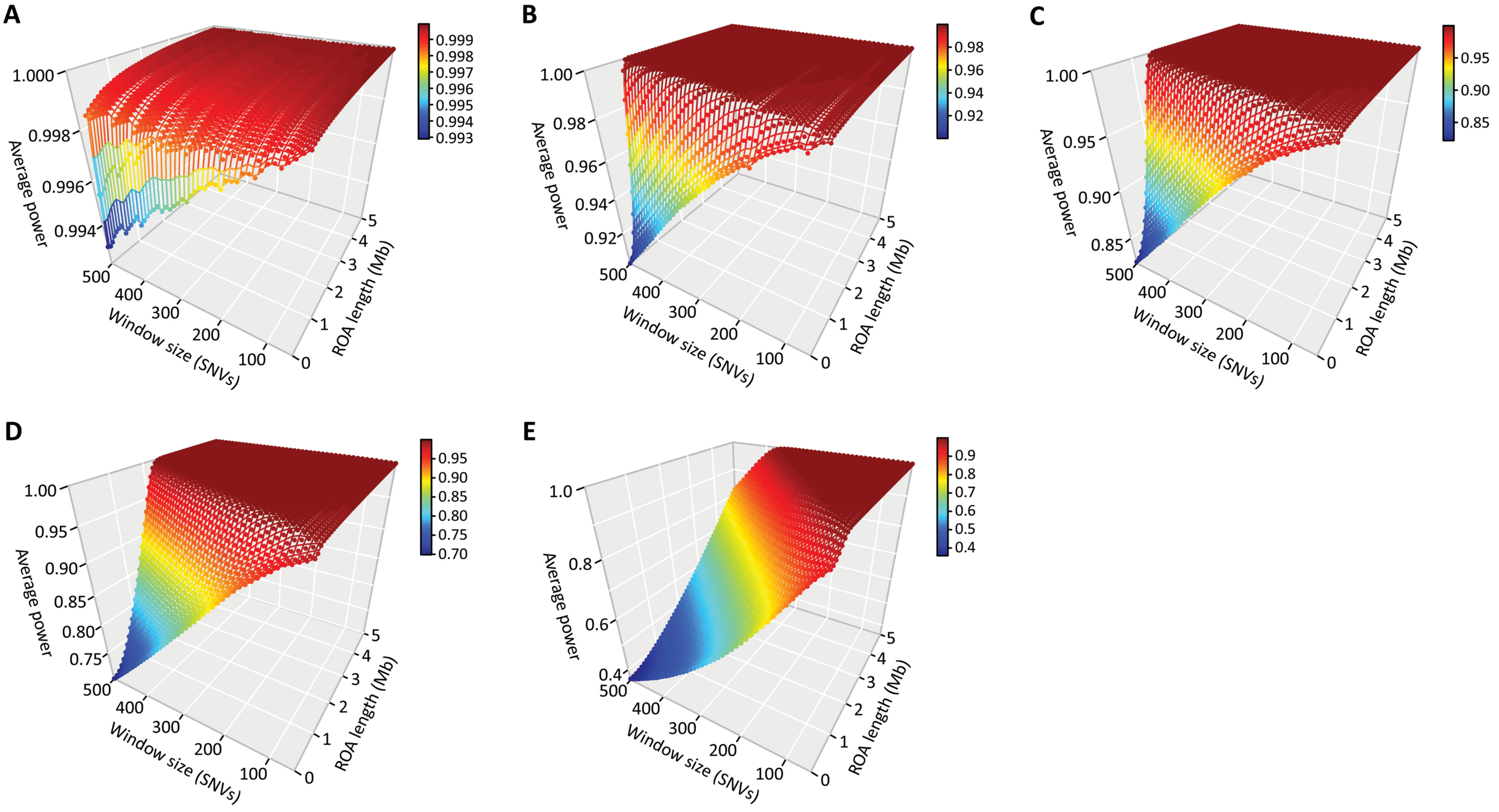

Figure S8. Effect of increasing window size on power to detect ROA of different lengths at different SNV densities. For scenario 1, 3D scatterplots depicting how window size affects power to detect ROA of different lengths averaged across the 50 replicates are shown separately for (**A**) 750,000 SNVs, (**B**), 125,000 SNVs, (**C**) 80,000 SNVs, (**D**) 50,000 SNVs, and (**E**) 18,000 SNVs. For each window size in each dataset, power was determined using either the smallest overlap fraction that had a ratio of inferred to true ROA length of ~1, or should all overlap fractions have a ratio less than one at that window size—an issue that arises at larger window sizes in lower density SNV datasets—then an overlap fraction of 0 was used.

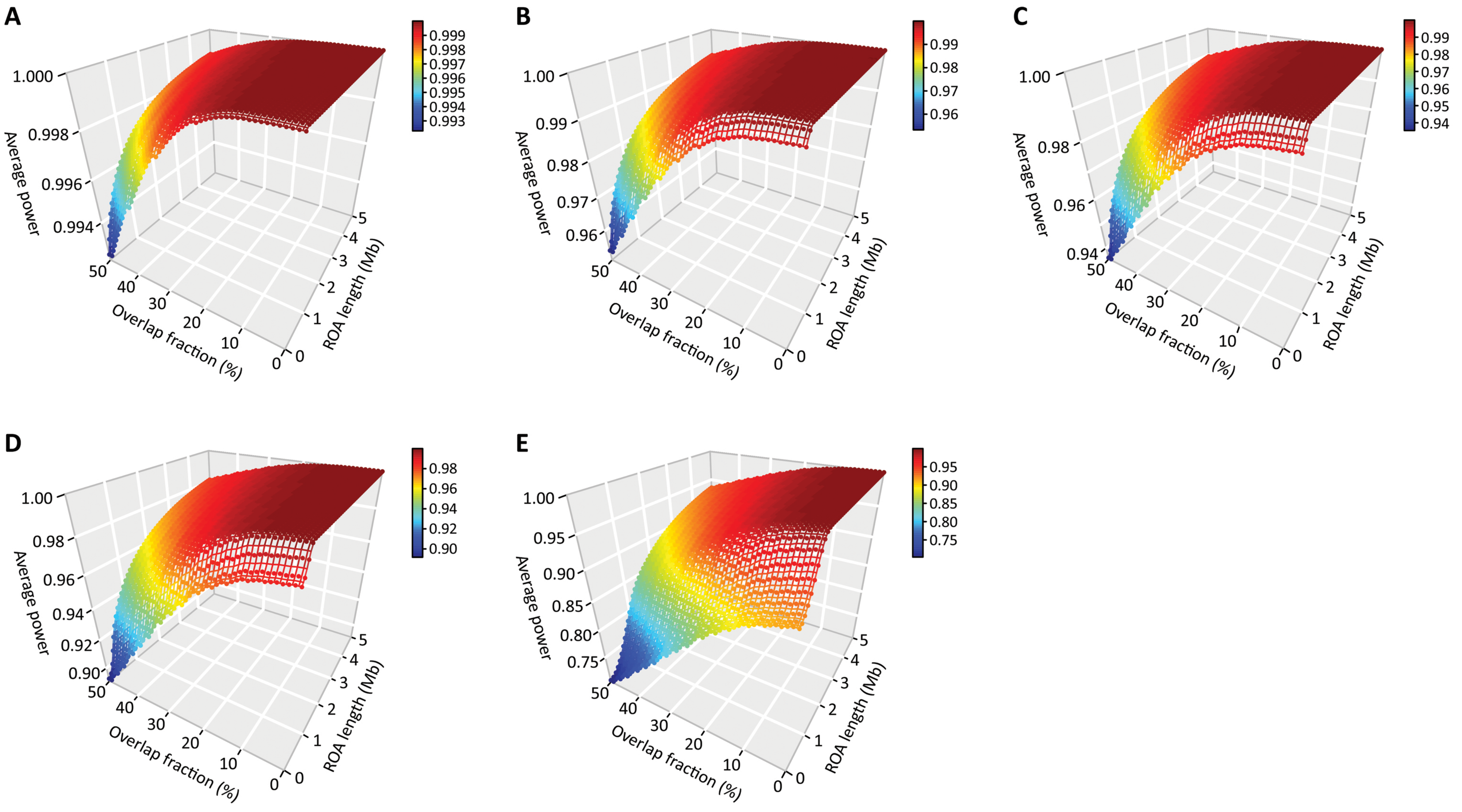

Figure S9. Effect of increasing overlap fraction on power to detect ROA of different lengths at different SNV densities. For scenario 1, 3D scatterplots depicting how overlap fraction affects power to detect ROA of different lengths averaged across the 50 replicates are shown separately for (**A**) 750,000 SNVs, (**B**), 125,000 SNVs, (**C**) 80,000 SNVs, (**D**) 50,000 SNVs, and (**E**) 18,000 SNVs. For each dataset power was evaluated at the optimal window size determined for that dataset (Additional File 2: **Table S1**).

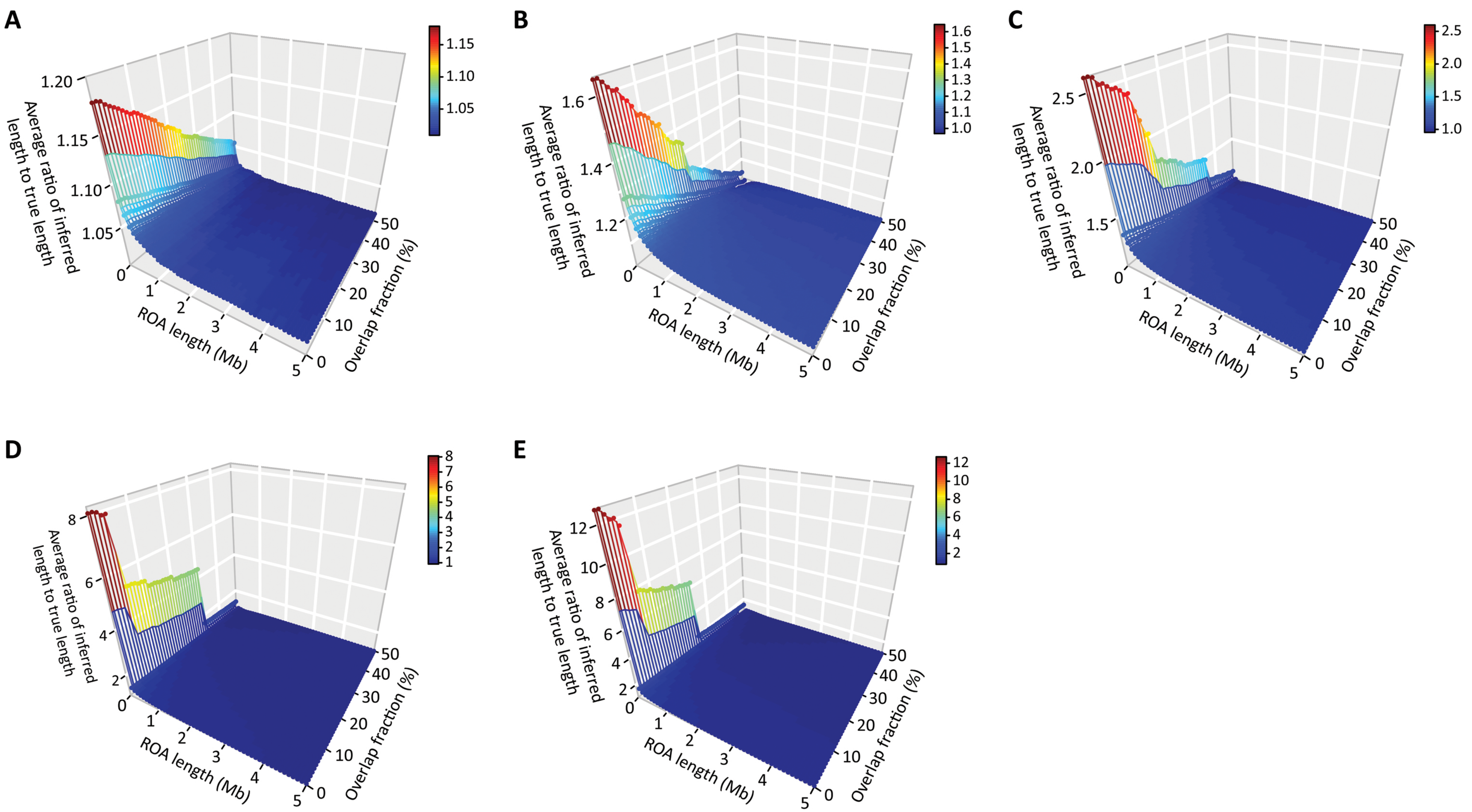

Figure S10. Effect of increasing overlap fraction on the ratio of inferred to true ROA length at different SNV densities. For scenario 1, 3D scatterplots depicting how overlap fraction affects the ratio of inferred to true length for ROA of different lengths averaged across the 50 replicates are shown separately for (**A**) 750,000 SNVs, (**B**), 125,000 SNVs, (**C**) 80,000 SNVs, (**D**) 50,000 SNVs, and (**E**) 18,000 SNVs. For each dataset power was evaluated at the optimal window size determined for that dataset (Additional File 2: **Table S1**).

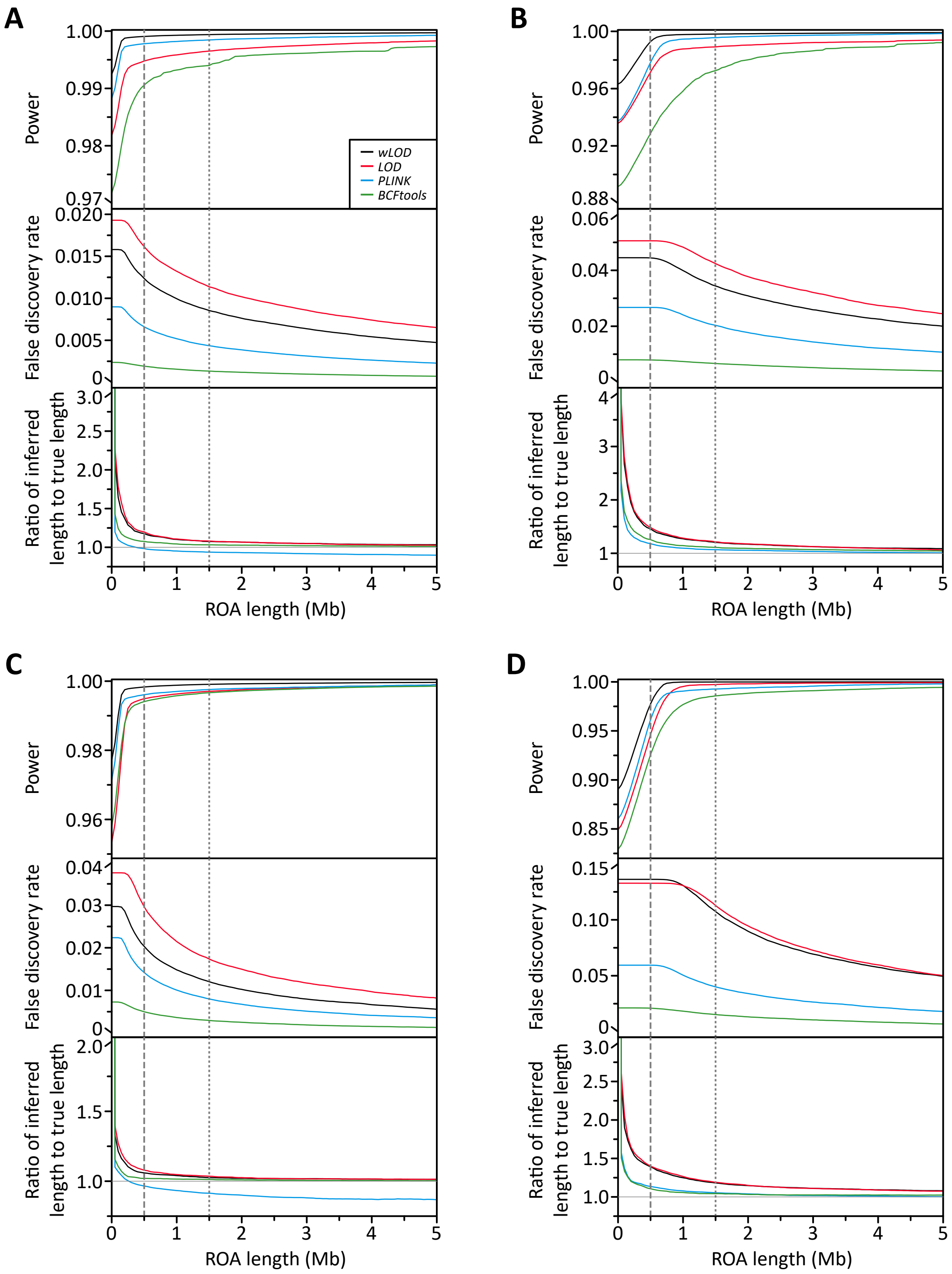

Figure S11. Performance of the *wLOD* method compared with existing methods. Line graphs showing for scenarios 1 (**A & B**) and 2 (**C & D**) and subsets consistent with the Illumina CanineHD BeadChip (18,000 SNV; **A & C**) and with the Illumina BovineHD BeadChip and Genome-Wide Human SNP 6.0 Microarray (70,000 SNV; **B & D**) how average power (top), false discovery rate (middle), and ratio of inferred and true ROA length (bottom) across 50 replicate genetic simulations change with increasing ROA length. The grey vertical lines denote 500 kb (dashed) and 1.5 Mb (dotted).

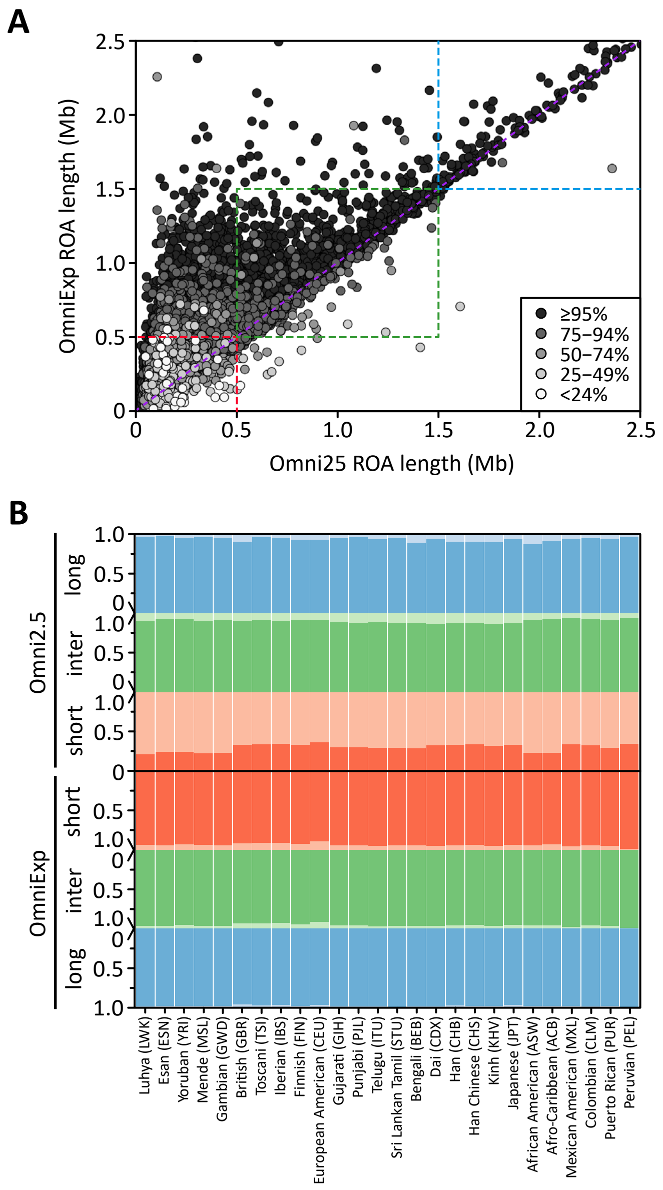

Figure S12. Concordance of ROA inferred in the Omni2.5 and OmniExp datasets. (**A**) A scatterplot comparing the length of each Omni2.5 ROA with that of its corresponding OmniExp ROA in the European American (CEU) population. Each point is shaded according to the proportion of the Omni2.5 ROA that overlaps the OmniExp ROA. (**B**) Bar plots representing the proportions of short (<500 kb; shown in red), intermediate (500 kb to 1.5 Mb; shown in green), and long (>1.5 Mb; shown in blue) ROA in the Omni2.5 (upper) and OmniExp (lower) datasets that overlap (darkest shade) or are absent from (lightest shade) the other dataset in each population. The figure follows the same format as **Figure 5**.

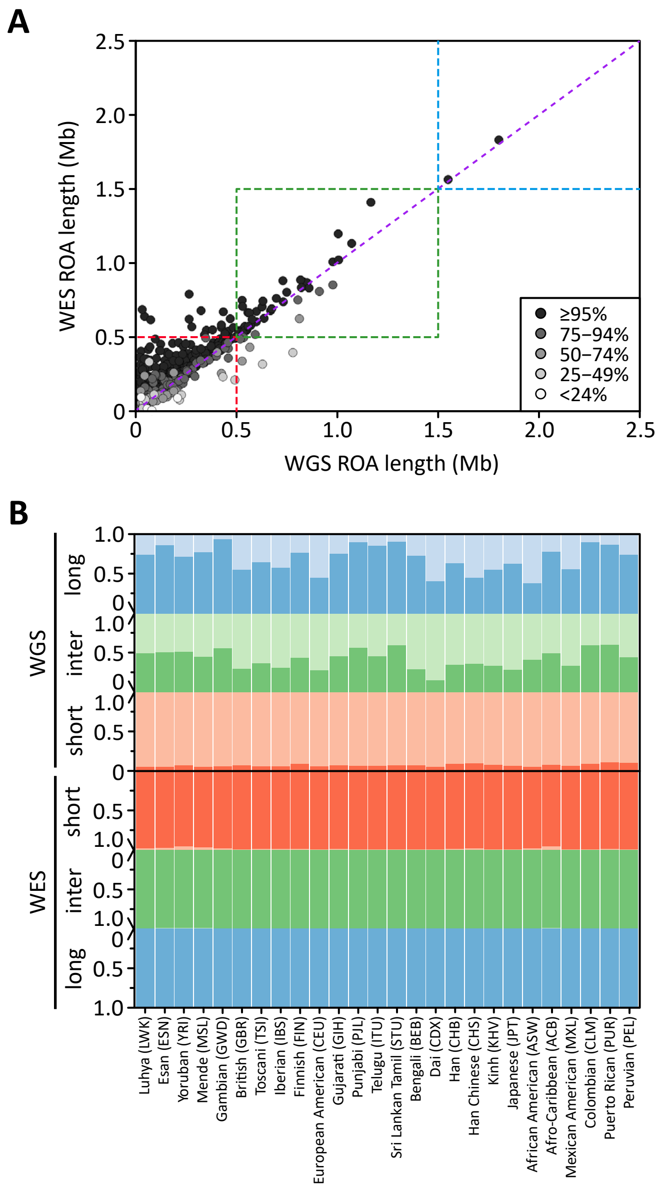

Figure S13. Concordance of ROA inferred in the WGS and WES datasets. (**A**) A scatterplot comparing the length of each WGS ROA with that of its corresponding WES ROA in the European American (CEU) population. Each point is shaded according to the proportion of the WGS ROA that overlaps the WES ROA. (**B**) Bar plots representing the proportions of short (<500 kb; shown in red), intermediate (500 kb to 1.5 Mb; shown in green), and long (>1.5 Mb; shown in blue) ROA in the WGS (upper) and WES (lower) datasets that overlap (darkest shade) or are absent from (lightest shade) the other dataset in each population. The figure follows the same format as **Figure 5**.

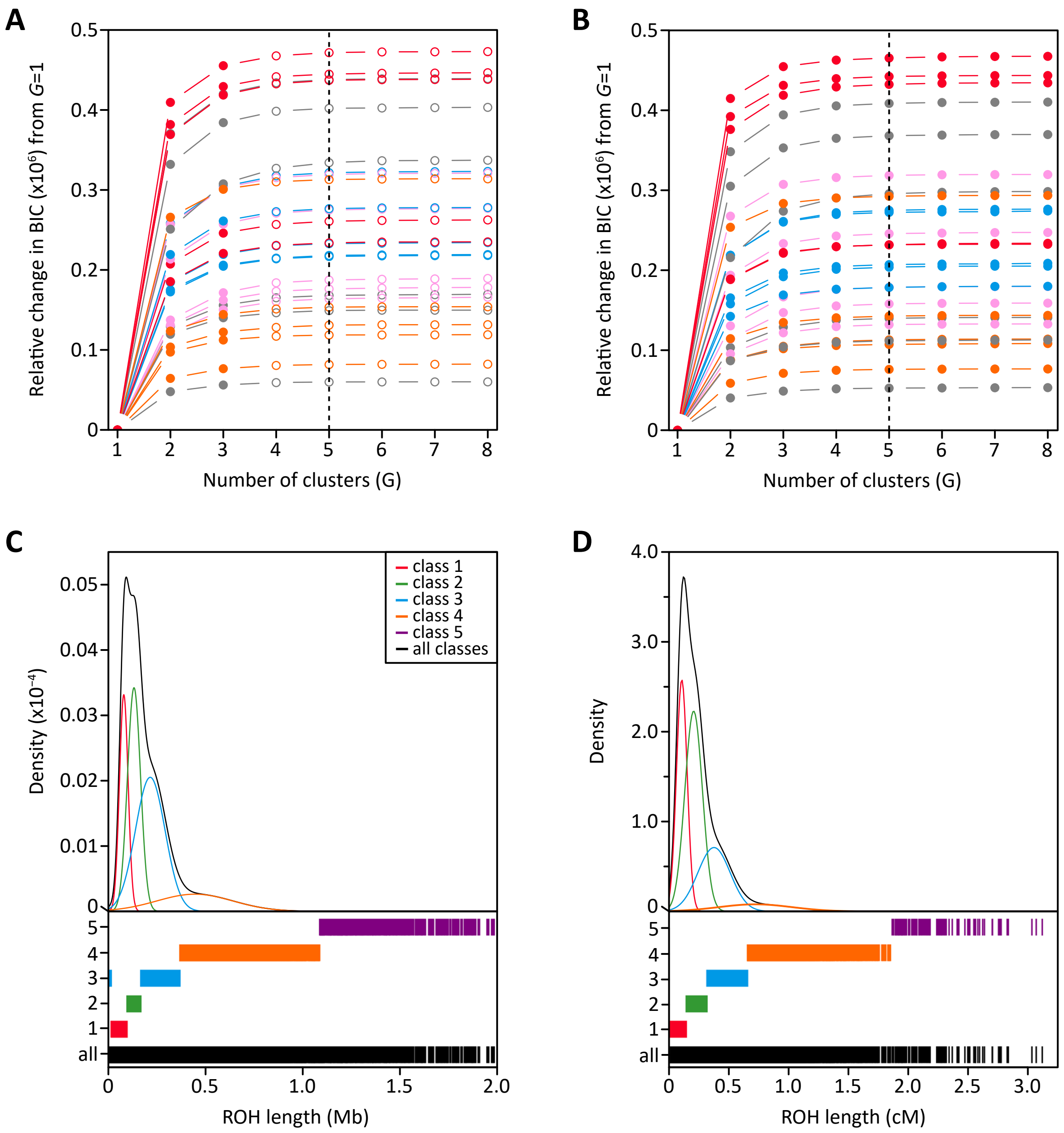

Figure S14. Properties of Gaussian mixture model classification of ROA. Line graphs showing the change in BIC relative to that obtained at *G*=1 with increasing number of clusters for Gaussian mixture model classifications based upon (**A**) physical map lengths and (**B**) genetic map lengths. Lines are colored by the geographic affiliation of the population as in **Figure S3** (Additional File 1). Filled circles denote values of *G* where all ROA classes are discrete, while open circles denote values of *G* where one or more classes were non-discrete (e.g. encompassed by another class). The black dashed line denotes *G*=5, the point at which the relative change in BIC plateaued for both physical and genetic map length. Example classifications of ROA identified in the CEU population based upon (**C**) physical map lengths and (**D**) genetic map lengths at *G*=5 are shown. Gaussian kernel density estimations of the ROA size distribution for all ROA (black line) and separately for each class are shown on top, with the inferred class assignments of ROA into each of the five classes shown below. Only ROA less than 2 Mb (in **C**) or 3.5 cM (in **D**) in length are shown, however, all ROA regardless of length were used in the analysis.

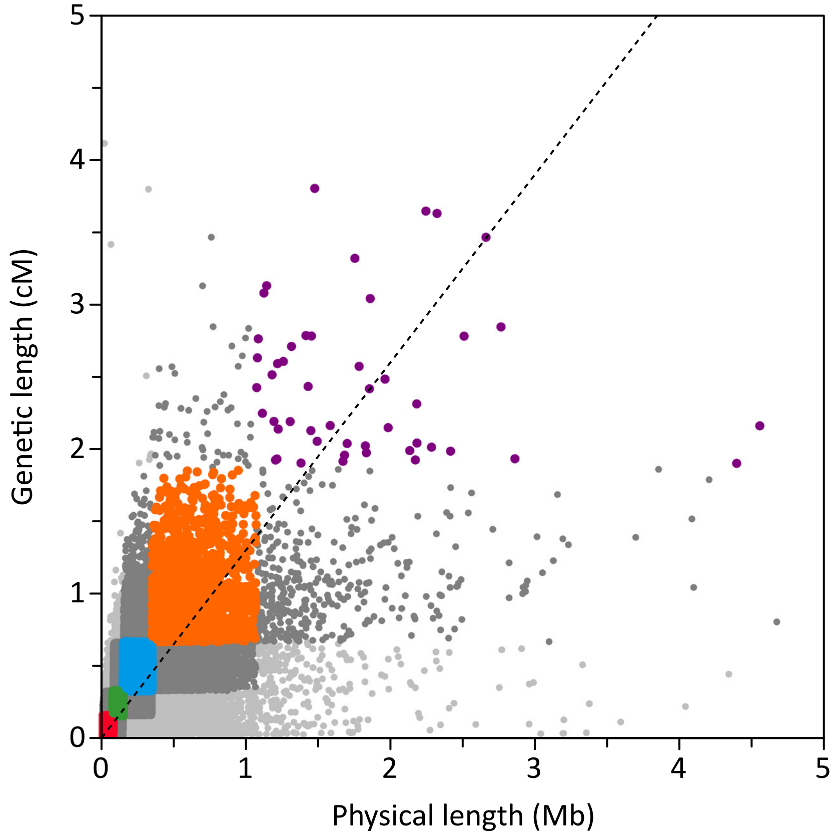

Figure S15. Correlation between physical and genetic map lengths of ROA. A scatterplot comparing the physical and genetic map lengths of all ROA identified in individuals from the CEU population with the Omni2.5 dataset. ROA placed in the same class when classified using their physical and genetic map lengths are shown in color as in **Figure S14C** (Additional File 1), ROA whose class differed by one step are shown in dark grey, and ROA whose class differed by more than one step are shown in light grey. The black dashed line depicts the commonly used conversion of 1.3 cM per Mb [242].

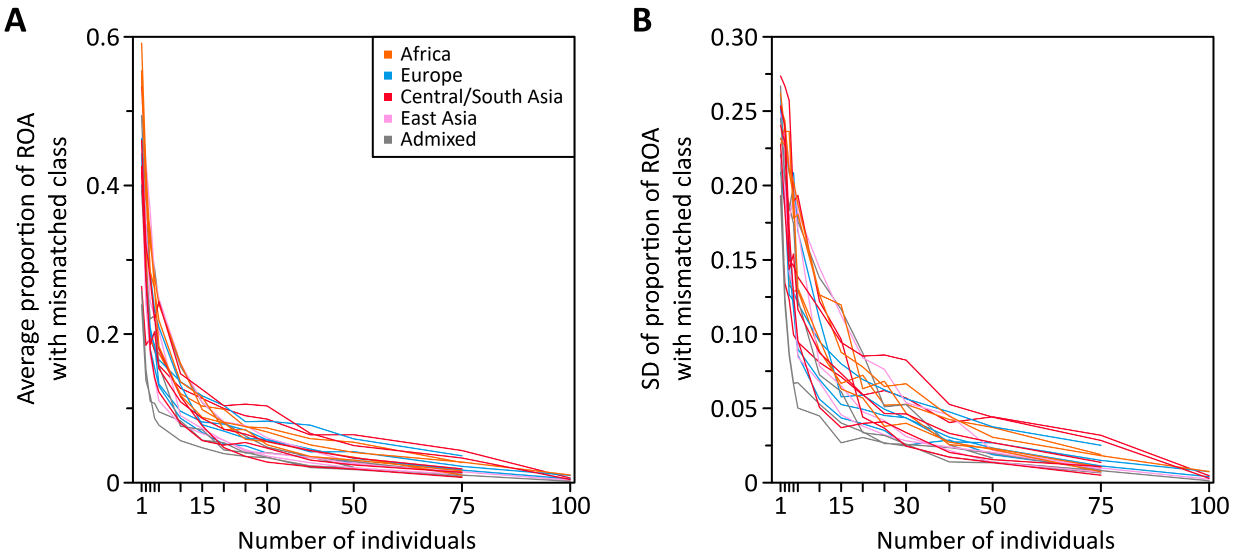

Figure S16. Effect of sample size on ROA length classification. Line graphs depicting (**A**) the average proportion of ROA whose classification at a given sample size differs from that obtained when all individuals are considered and (**B**) its standard deviation across 100 randomly-sampled replicates for the Omni2.5 dataset. Lines are colored by the geographic affiliation of the population: Africa, orange; Europe, blue; Central/South Asia, red; East Asia, pink; admixed, grey.

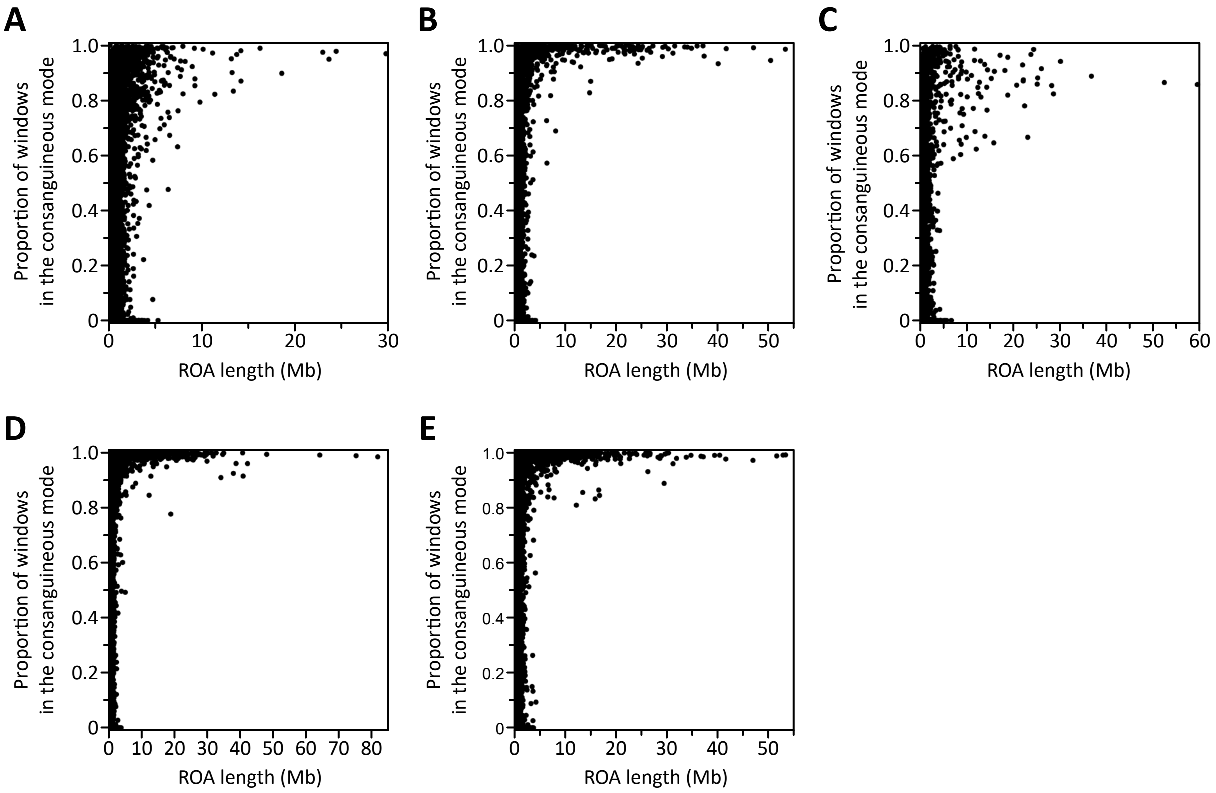

Figure S17. Effect of ROA length on the proportion of windows drawn from the consanguinity autozygous mode. Scatterplots showing for each ROA the proportion of its component windows that had $wLOD$ scores in the putative consanguinity-associated autozygous mode plotted against its length shown separately for the (**A**) GIH (Spearman's ρ=0.234, $P<10^{-16}$), (**B**) ITU (ρ=0.261, $P<10^{-16}$), (**C**) CDX (ρ=0.185, $P<10^{-16}$), (**D**) PJL (ρ=0.302, $P<10^{-16}$), and (**E**) STU (ρ=0.286, $P<10^{-16}$) populations.

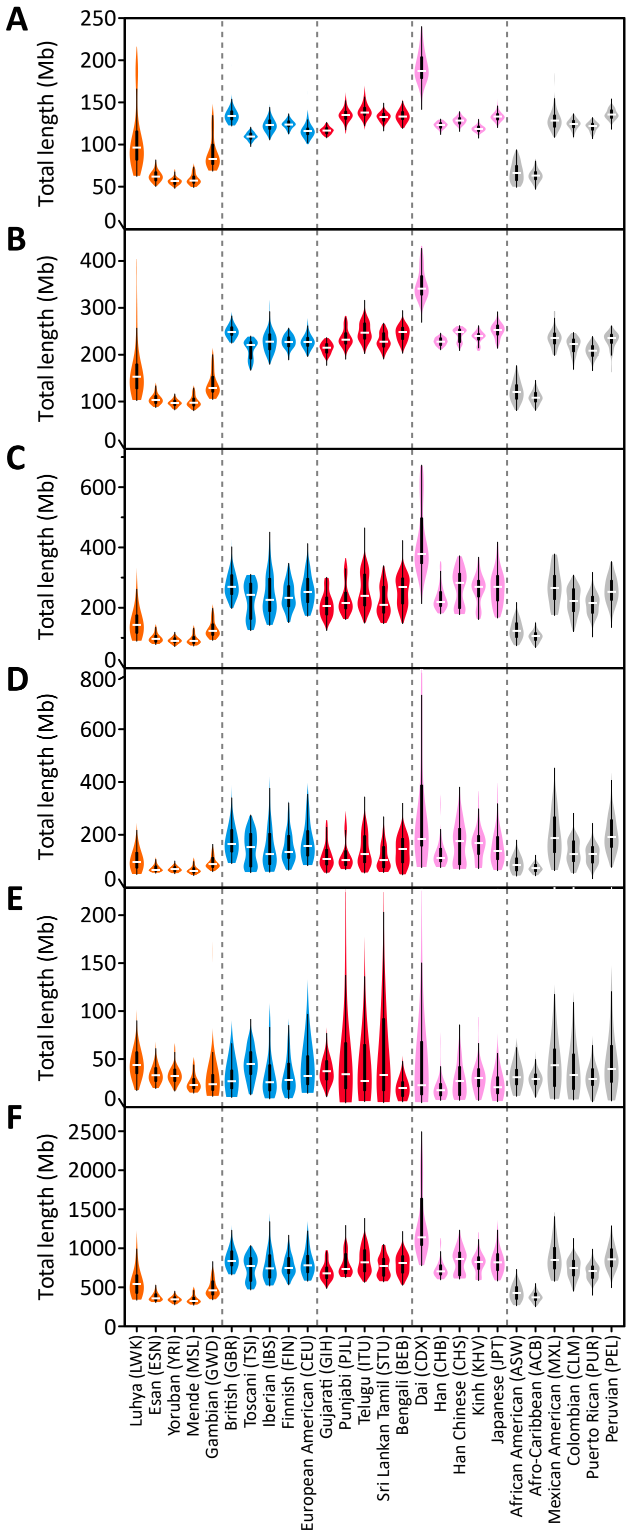

Figure S18. Population-specific distributions of the total length of ROA per individual. Data are shown as violin plots [252], representing the distribution of total ROA length across all individuals in each of the 26 populations for (**A**) class 1, (**B**) class 2, (**C**) class 3, (**D**) class 4, (**E**) class 5, and (**F**) all five ROA classes combined. Populations are ordered from left to right by geographic region and within each region by increasing geographic distance from Addis Ababa.

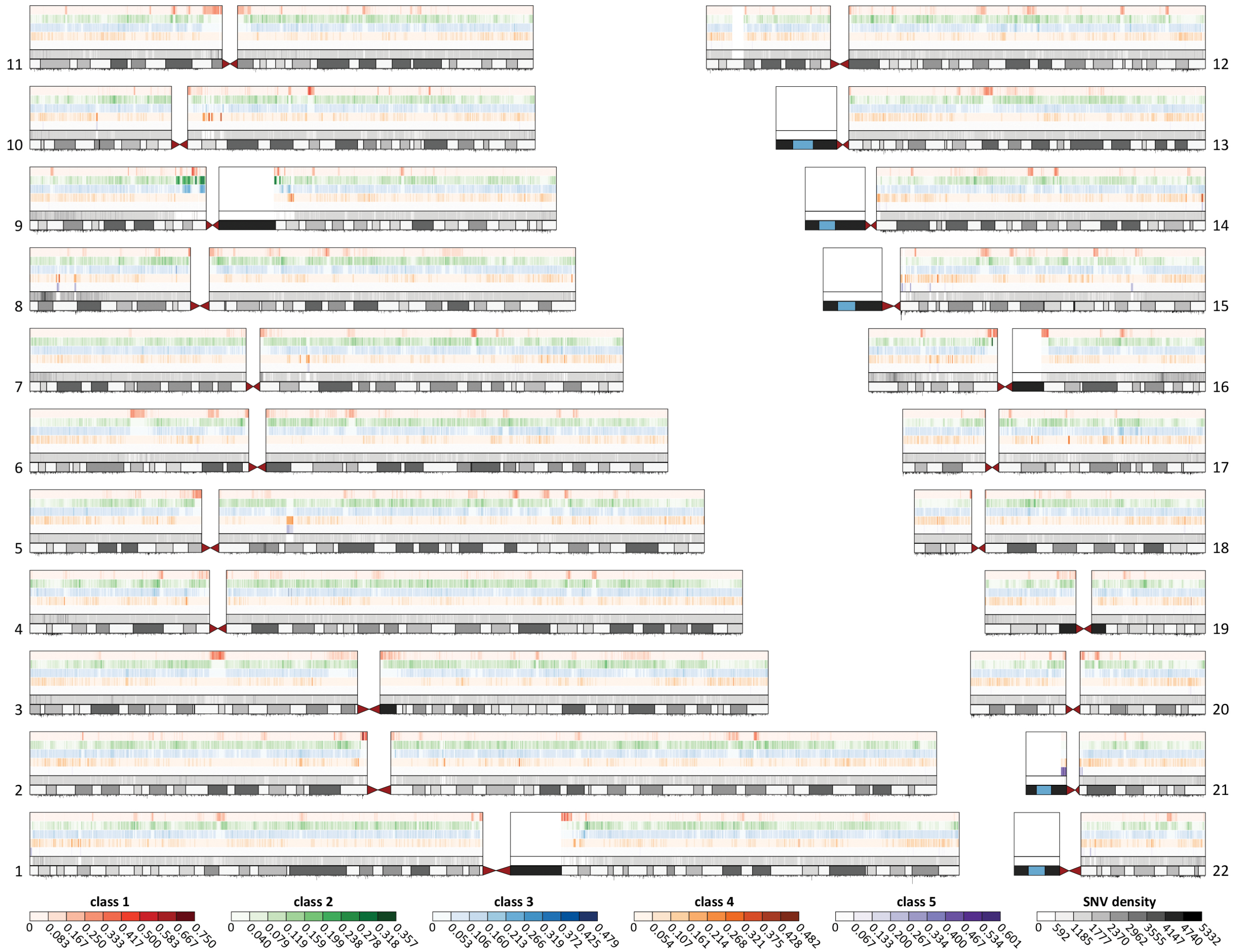

Figure S19. Distribution of worldwide ROA frequencies across the genome. For each autosome, the figure shows for each ROA length class the average proportion of individuals in the WGS dataset who have an ROA overlapping SNVs within non-overlapping 50 kb windows. Each row represents an ROA class, and each column represents a window. The intensity of a point increases with increasing average ROA frequency, as indicated by the color scale below figure. The SNV density of each window and an ideogram of chromosome banding are shown in the bottom tracks, with average recombination rate in each window represented by a vertical black line below the ideogram, where line heights proportional to average recombination rate.

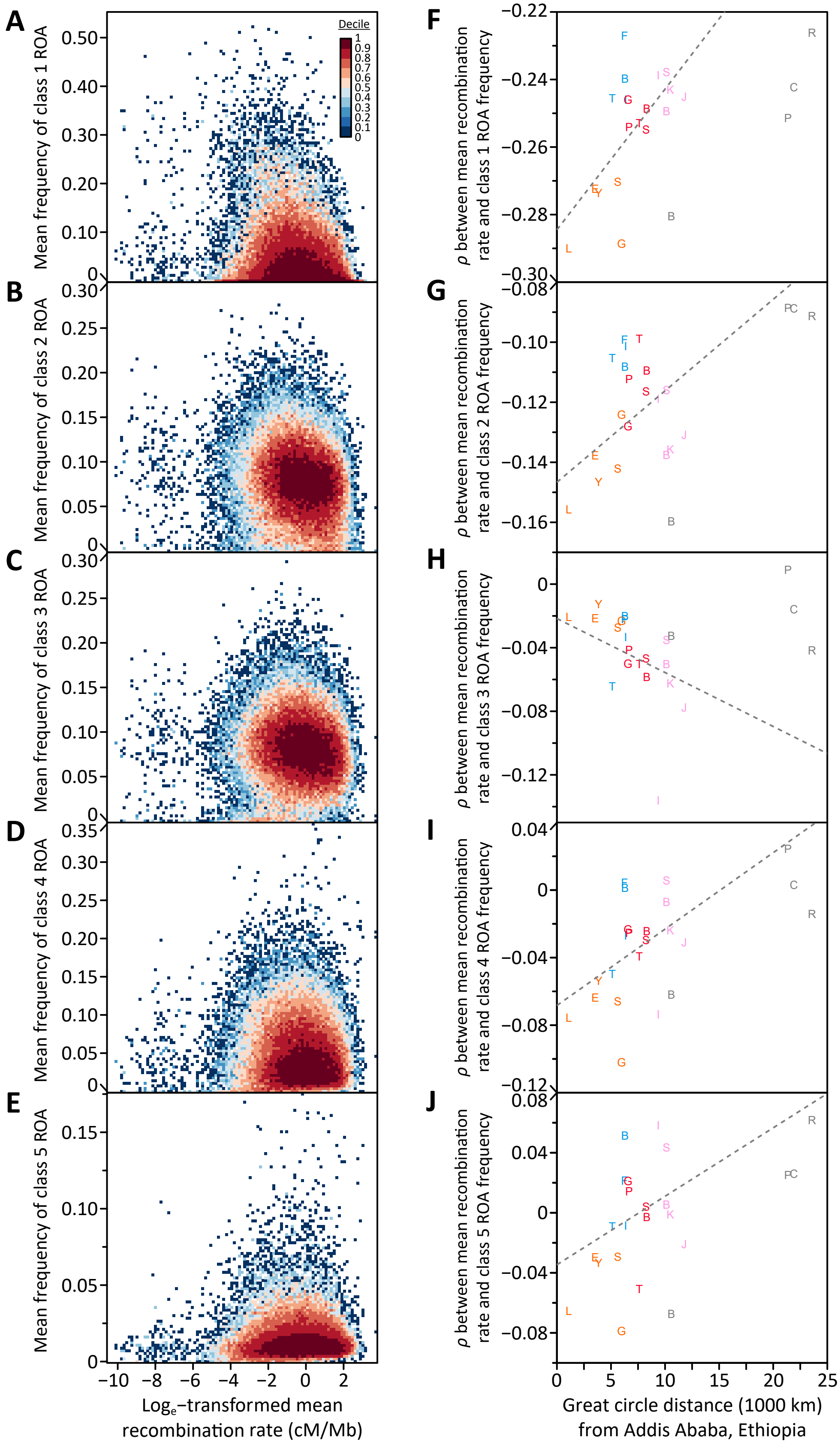

Figure S20. Relationship between ROA frequencies and recombination rate. Heat maps comparing average worldwide ROA frequency in the WGS dataset and recombination rate in non-overlapping 50 kb windows separately for (**A**) class 1 (ρ=-0.271, P<10^{-16}), (**B**) class 2 (ρ=-0.157, P<10^{-16}), and (**C**) class 3 (ρ=-0.084, P<10^{-16}), (**D**) class 4 (ρ=-0.040, P<10^{-16}), and (**E**) class 5 (ρ=0.034, P<10^{-16}) ROA. Cells are colored by decile. Scatterplots comparing the Spearman's rank correlation ρ between average per-population ROA frequency and recombination rate in non-overlapping 50 kb windows with geographic distance from Addis Ababa are shown for (**F**) class 1 (ρ=-0.638, P=0.002), (**G**) class 2 (ρ=0.260, P=0.141), and (**H**) class 3 (ρ=-0.597, P=0.003), (**I**) class 4 (ρ=0.469, P=0.021), and (**J**) class 5 (ρ=0.464, P=0.023) ROA. Populations are indicated by the same symbols and colors as in **Figure S24** (Additional File 1). The number of data points (non-overlapping windows) examined was 52,425.

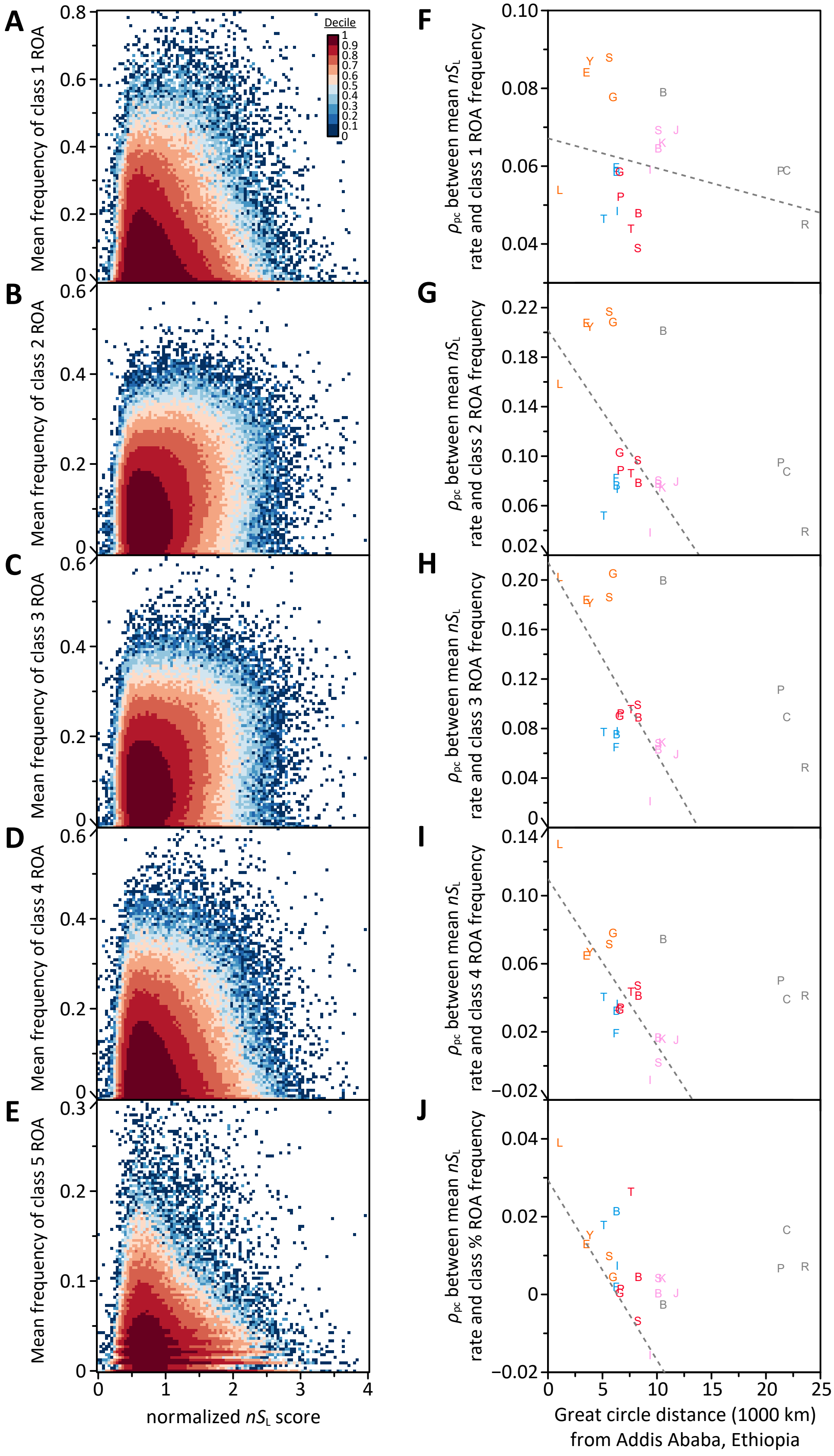

Figure S21. Relationship between ROA frequencies and nS_L selection scores. Heat maps comparing average per-population ROA frequency in the WGS dataset and nS_L score in non-overlapping 50 kb windows are shown separately for (**A**) class 1 (ρ_{pc}=-0.043, P<10^{-16}), (**B**) class 2 (ρ_{pc}=-0.170, P<10^{-16}), (**C**) class 3 (ρ_{pc}=-0.199, P<10^{-16}), (**D**) class 4 (ρ_{pc}=-0.105, P<10^{-16}), and (**E**) class 5 (ρ_{pc}=-0.007, P=3.16×10^{-14}) ROA. Averages were calculated separately for each population and then pooled to create the heat map. Cells are colored by decile. Scatterplots comparing the Spearman's partial rank correlation ρ_{pc} between average per-population ROA frequency and nS_L score in non-overlapping 50 kb windows with geographic distance from Addis Ababa are shown for (**F**) class 1 (ρ=-0.140, P=0.716), (**G**) class 2 (ρ=-0.607, P=0.003), (**H**) class 3 (ρ=-0.741, P=1.44×10^{-4}), (**I**) class 4 (ρ=-0.824, P=7.37×10^{-6}), and (**J**) class 5 (ρ=-0.662, P=0.001) ROA. All ρ_{pc} had P<0.002. Populations are indicated by the same symbols and colors as in **Figure S24** (Additional File 1). The number of data points (non-overlapping windows) examined was 52,425.

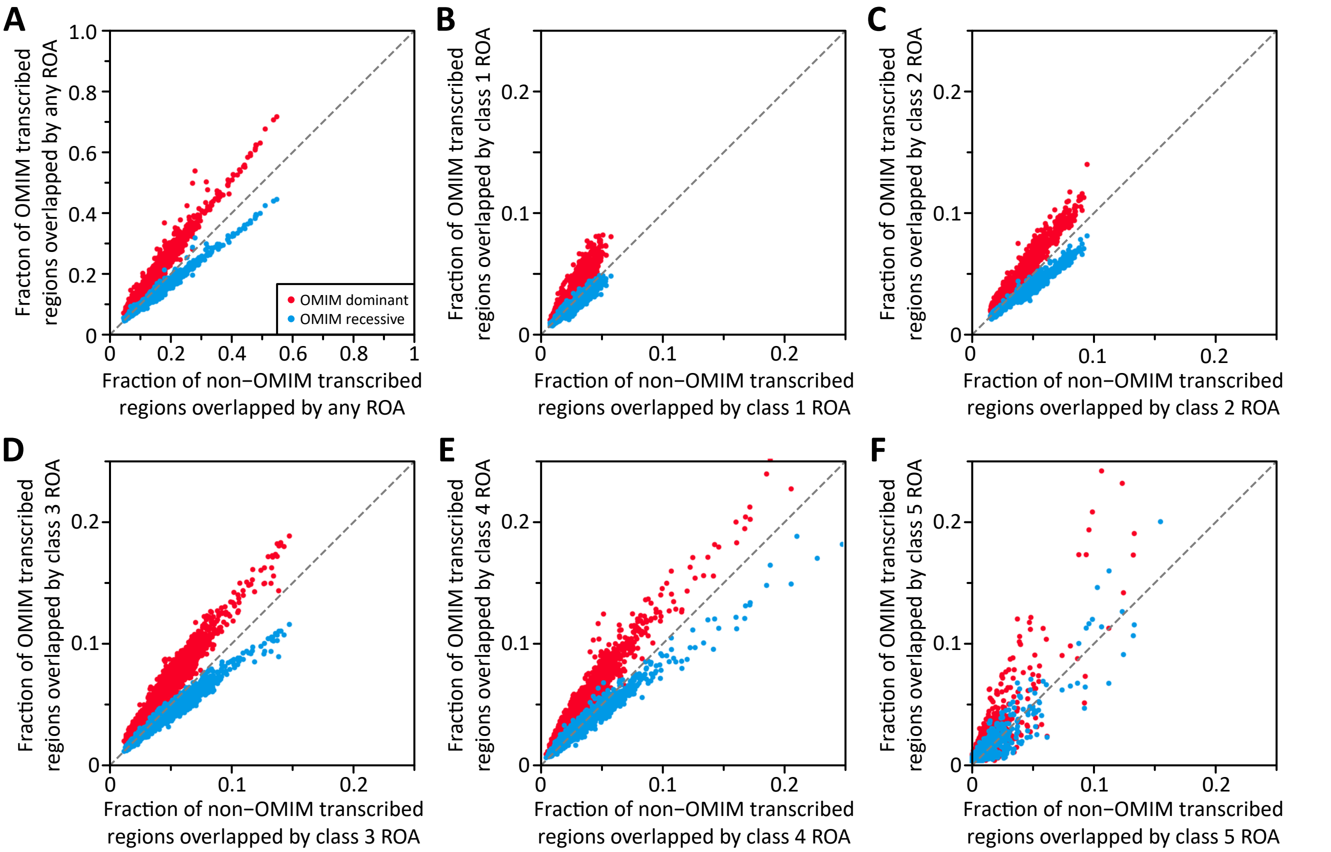

Figure S22. Fraction of OMIM autosomal recessive and dominant gene regions in ROA relative to non-OMIM genes. Scatterplots comparing the fraction of the total length of all transcribed regions of OMIM dominant (red) and OMIM recessive (blue) genes relative to the fraction for non-OMIM genes in each individual's genome, shown separately for (**A**) all ROA, (**B**) class 1 ROA, (**C**) class 2 ROA, (**D**) class 3 ROA, (**E**) class 4 ROA, and (**F**) class 5 ROA. The identity line is shown in grey.

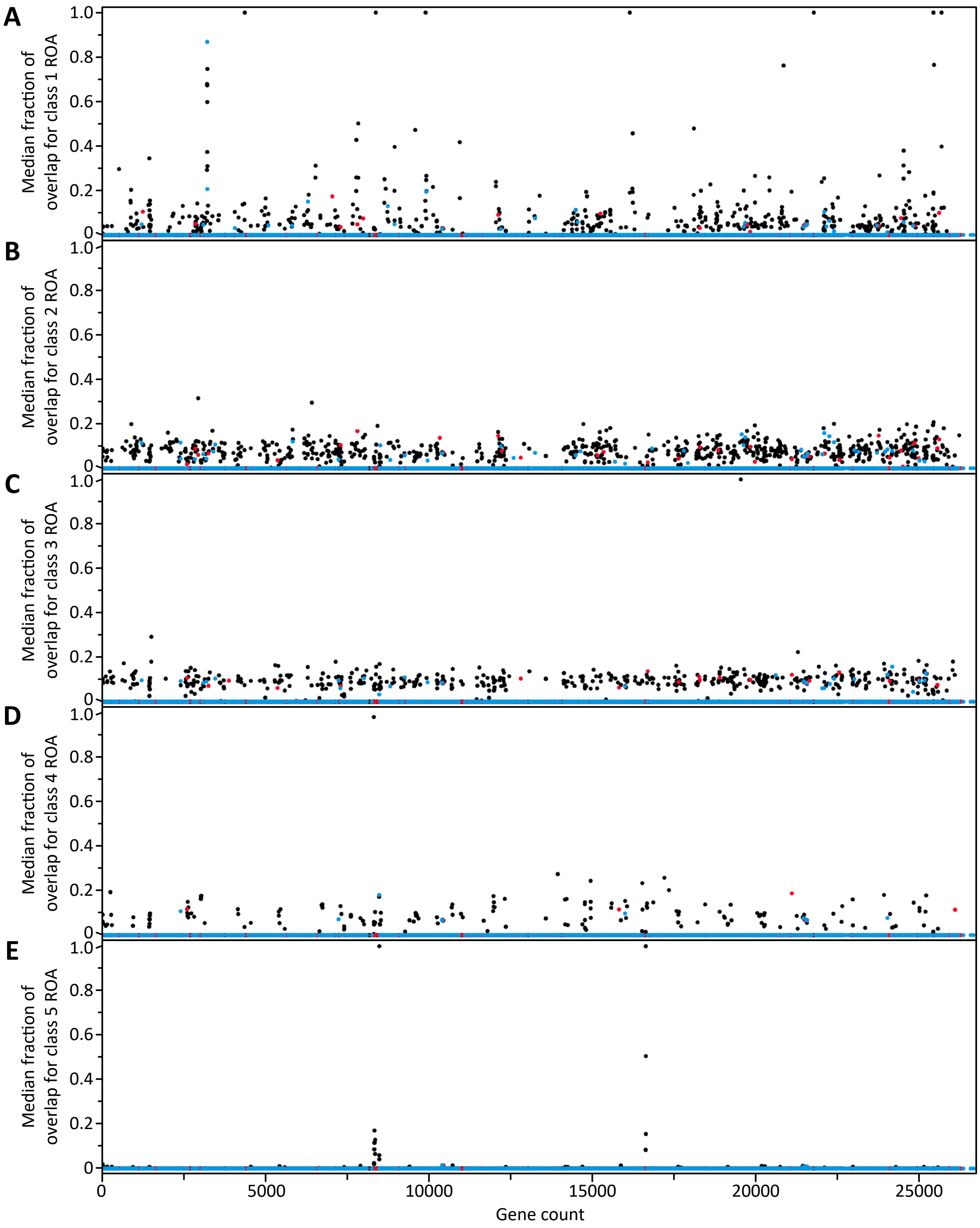

Figure S23. Median fraction of each autosomal gene in ROA in the CEU population. Manhattan plots depicting for each autosomal gene the median fraction across individuals in the Northern European CEU population of its transcribed region that overlaps a ROA, shown separately for (**A**) class 1 ROA, (**B**) class 2 ROA, (**C**) class 3 ROA, (**D**) class 4 ROA, and (**E**) class 5 ROA. OMIM recessive genes are in red, OMIM dominant genes are in blue, and non-OMIM genes are in back. Genes are ordered from left to right by increasing position in the genome.

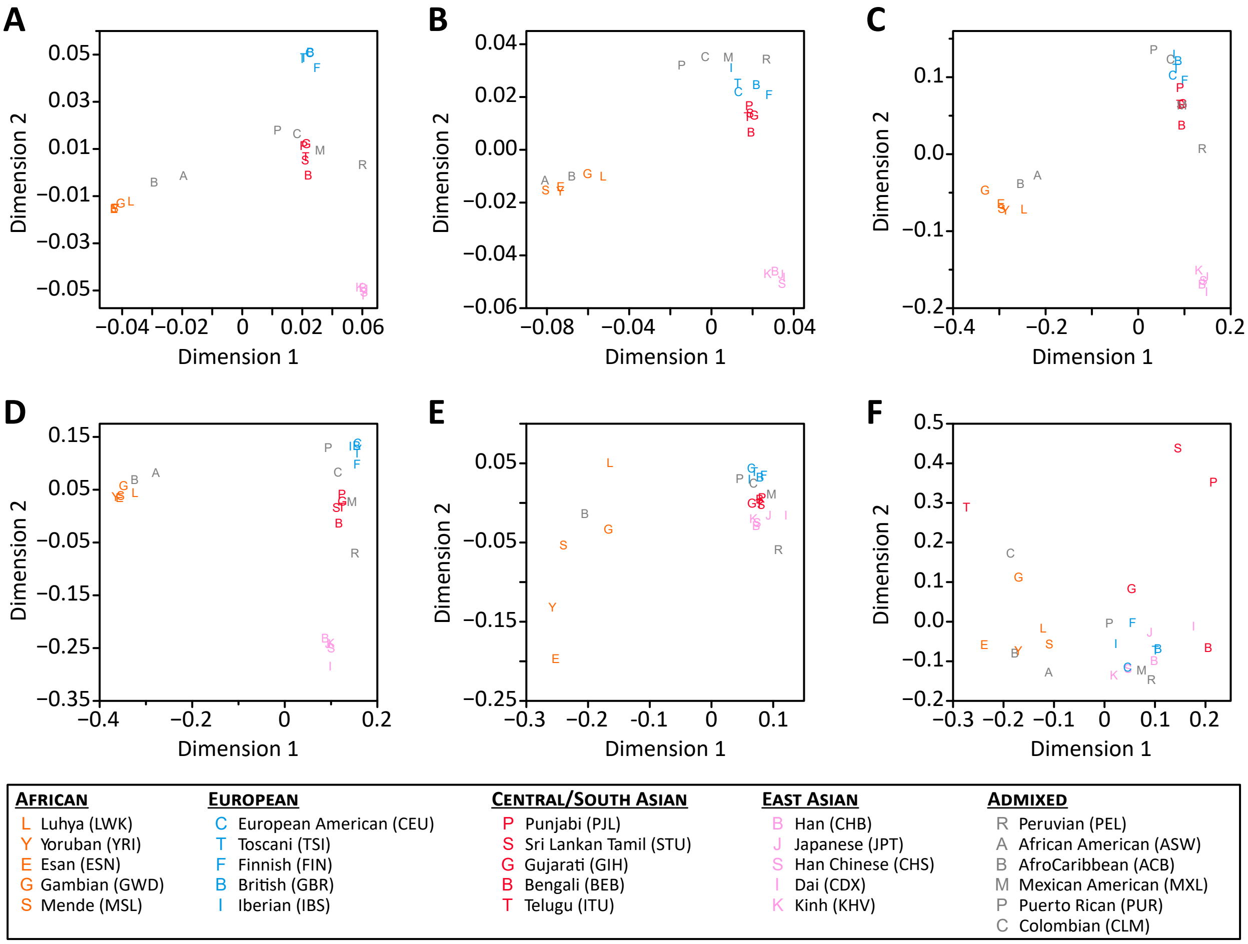

Figure S24. Geographic Groupings in the genomic distribution of ROA. Procrustes-transformed [250] multidimensional scaling (MDS) representations of (**A**) pairwise F_{ST} among populations and of pairwise correlations between genome-wide ROA frequencies in individual populations shown separately for (**B**) class 1 (Procrustes similarity statistic t_0=0.969), (**C**) class 2 (t_0=0.939), (**D**) class 3 (t_0=0.973), (**E**) class 4 (t_0=0.803), and (**F**) class 5 (t_0=0.466) ROA. All computations considered SNV and ROA frequencies in the WGS dataset.

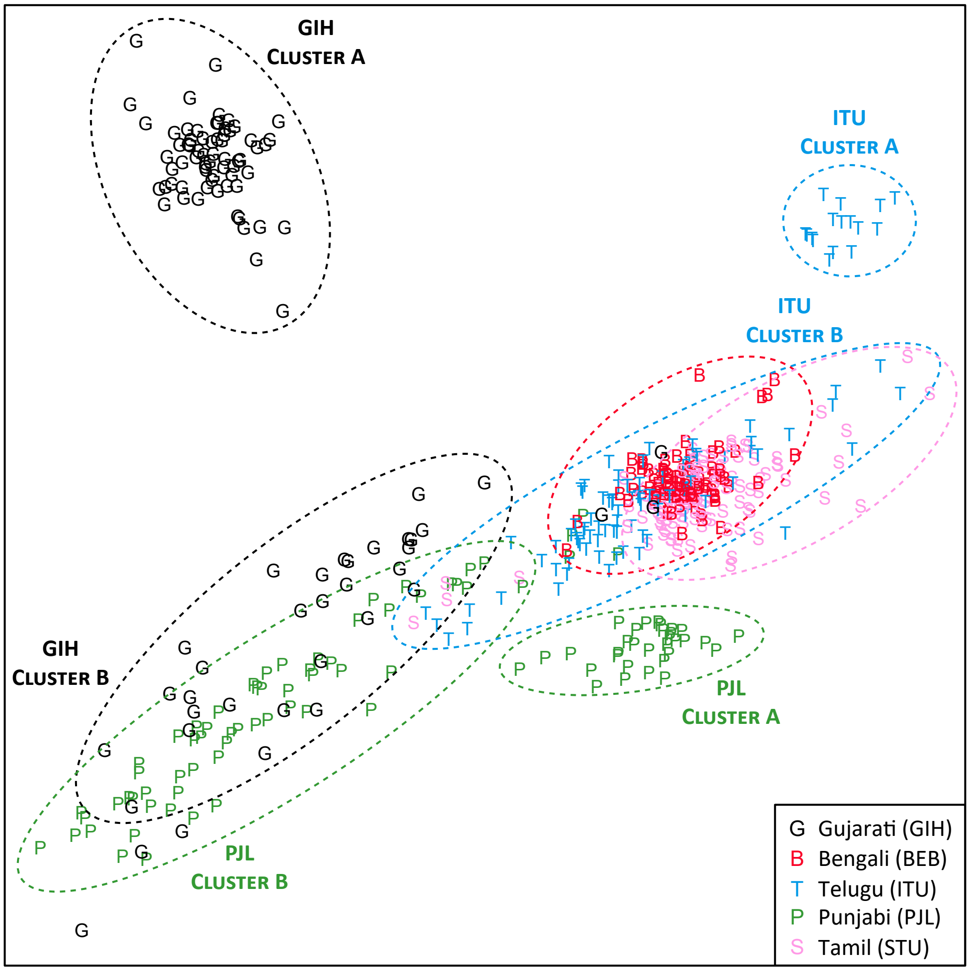

Figure S25. Evidence of genetic structure within three Asian Indian groups. A multidimensional scaling (MDS) representation of pairwise allele sharing dissimilarities (ASD) among individuals in the five Asian Indian groups. Subgroupings of individuals in the GIH, ITU, and PJL groups are labeled. The pairwise ASD matrix was constructed with *asd* (https://github.com/szpiech/asd) considering only the Asian Indian individuals and MDS applied to this matrix using *cmdscale* in *R*.

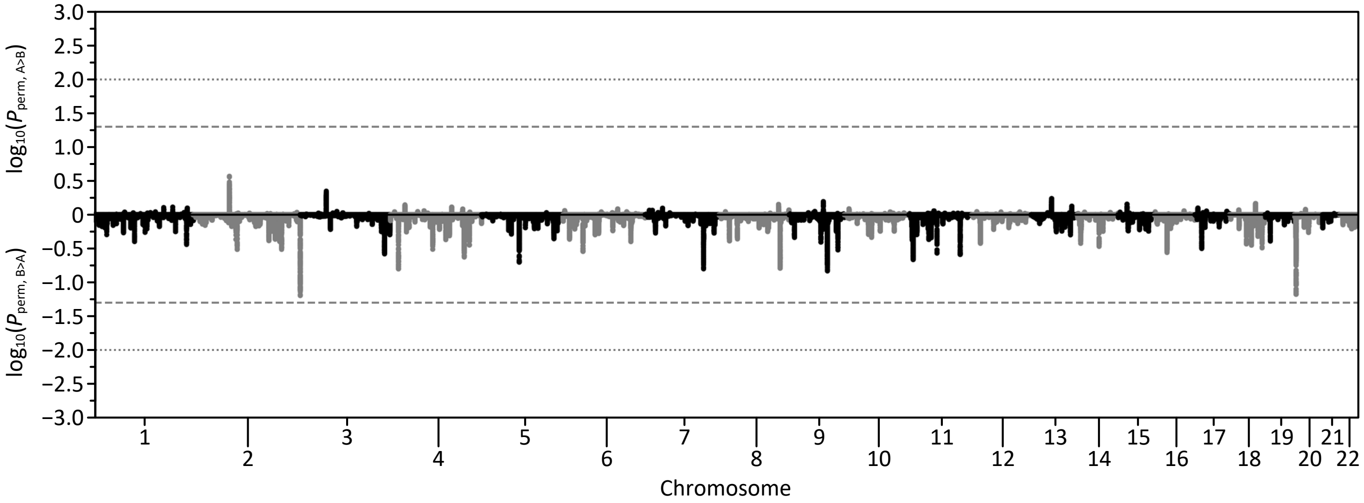

Figure S26. Distribution of differential ROA signals between subgroups in the GIH. Manhattan plot showing for each window the $log_{10}(P)$ of pairwise comparisons of per-individual $wLOD$ scores in the two subgroups present in the GIH (450 SNV window). The figure follows the same format as **Figure 8**.

Additional File 1: Weighted Likelihood Inference of Autozygosity

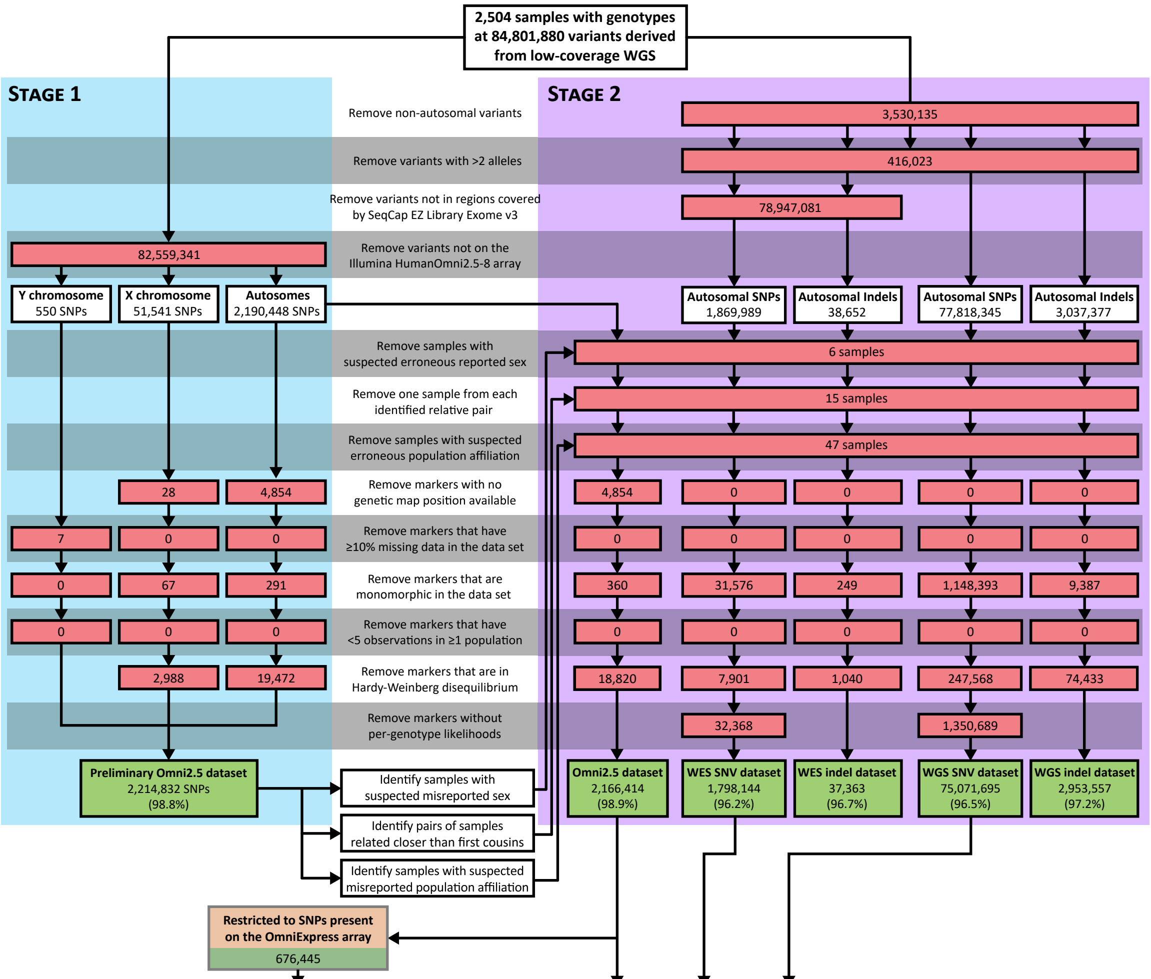

Number of Polymorphic SNPs: OmniExpress dataset		Number of Polymorphic SNPs: Omni2.5 dataset	WES SNV dataset	WGS SNV dataset	Restricted to SNPs with MAF ≥ 0.05: Omni dataset	WES SNV dataset	WGS SNV dataset
659,635	African Americans (ASW)	1,875,703	259,937	15,678,610	1,470,129	123,752	8,271,798
609,502	European Americans (CEU)	1,504,314	157,475	9,329,585	1,229,672	85,969	5,763,441
573,796	Northern Han Chinese (CHB)	1,295,974	149,329	8,561,123	1,101,550	78,897	5,291,131
612,061	Gujarati Asian Indians (GIH)	1,444,496	167,430	9,725,875	1,215,783	88,386	5,924,502
566,532	Japanese (JPT)	1,276,110	143,443	8,257,847	1,098,103	79,102	5,296,946
637,505	Luhya (LWK)	1,783,880	273,423	16,214,885	1,423,404	125,205	8,412,802
628,651	Mexican Americans (MXL)	1,550,183	169,206	10,107,072	1,200,136	85,921	5,780,327
612,769	Toscani (TSI)	1,490,792	165,916	9,655,036	1,225,097	86,357	5,789,111
627,712	Yorubans (YRI)	1,820,331	258,055	15,496,462	1,453,506	126,038	8,424,495
607,625	Finnish (FIN)	1,461,487	147,463	8,772,002	1,211,332	86,391	5,764,322
607,603	British Europeans (GBR)	1,474,211	153,764	9,095,552	1,220,089	86,075	5,756,002
618,184	Iberian (IBS)	1,522,838	168,315	9,860,003	1,227,909	86,392	5,796,190
569,170	Southern Han Chinese (CHS)	1,277,537	145,872	8,355,628	1,095,376	78,308	5,267,566
639,746	Colombian (CLM)	1,632,025	186,880	11,163,131	1,253,526	89,793	6,027,057
649,753	Puerto Rican (PUR)	1,714,689	206,308	12,301,518	1,292,307	93,496	6,262,140
569,155	Dai (CDX)	1,273,263	144,103	8,344,783	1,088,441	78,601	5,273,001
612,856	Peruvian (PEL)	1,457,729	154,025	9,213,811	1,084,462	77,100	5,206,300
576,888	Kinh (KHV)	1,298,906	150,021	8,714,738	1,099,137	79,211	5,312,057
654,725	Afro-Carribean (ACB)	1,876,566	273,367	16,326,314	1,465,237	125,390	8,397,764
614,693	Bengali Asian Indians (BEB)	1,453,857	177,486	10,141,487	1,219,704	87,729	5,898,949
625,358	Esan (ESN)	1,780,196	253,820	15,310,009	1,431,730	126,547	8,445,931
633,026	Gambian (GWD)	1,772,779	260,870	15,703,622	1,419,986	125,052	8,372,826
611,636	Telugu Asian Indians (ITU)	1,436,109	172,783	10,010,646	1,214,346	88,052	5,916,551
626,626	Mende (MSL)	1,768,757	265,028	15,930,048	1,427,775	128,314	8,588,370
615,350	Punjabi Asian Indians (PJL)	1,463,840	173,959	10,113,064	1,228,563	88,566	5,944,126
611,985	Sri Lankan (STU)	1,435,924	173,077	10,037,651	1,214,686	88,109	5,914,004

Figure S27. Flow diagram of the sequential data filtering steps used for preparation of the datasets used in the analyses. Steps are shown in the order in which they were applied. SNVs that failed in one step were removed and were not considered in subsequent steps. The numbers of SNVs removed at each step are shown in the boxes shaded in red, the final numbers of SNVs in each dataset are shown in the boxes shaded in green.
