# Supplementary material for: Weighted likelihood inference of genomic autozygosity patterns in dense genotype data
Source: BMC Genomics. 2017 Dec 1;18:928. doi: 10.1186/s12864-017-4312-3 (PMC5709839; doi:10.1186/s12864-017-4312-3)
Supplement: Supplementary file 2 — Supplementary tables and their footnotes. (PDF 867 kb) [file 12864_2017_4312_MOESM2_ESM.pdf]

Additional File 2: Weighted Likelihood Inference of Autozygosity

Table S1. Optimal window size and overlap proportion with the $wLOD$ method in the simulated datasets.

SNV subset	Scenario 1		Scenario 2	
	Window size	% overlap	Window size	% overlap
18,000	60	7	70	5
50,000	70	18	80	19
80,000	80	28	90	22
125,000	80	29	100	26
750,0000	130	37	120	32

Table S2. Optimal window sizes and overlap fractions with the LOD method in the simulated datasets.

SNV subset	Scenario 1		Scenario 2	
	**Window size**	**% overlap**	**Window size**	**% overlap**
18,000	50	15	60	2
50,000	60	19	70	11
80,000	60	21	60	17
125,000	60	21	70	16
750,0000	80	14	80	17

Table S3. Genomic regions encompassing genes with a median fraction of overlap with class 5 ROA of > 0.9 in at last one population.

Genomic region			Genes	Populations
Chr	Begin (bp)	End (bp)		
8	7,118,140	7,143,880	*LINC00965*	ASW, LWK
8	7,627,105	7,628,835	*FAM90A10P*	LWK, TSI
15	28,818,925	28,834,386	*HERC2P11*	CEU
21	9,825,831	9,826,263	*MIR3648, MIR3687*	CEU, JPT, LWK, TSI, GBR, CDX, GWD
21	9,907,188	9968594	*TEKT4P2*	CDX
21	11,020,841	11,098,937	*BAGE, BAGE2, BAGE3, BAGE4, BAGE5*	TSI, GBR, IBS, CLM, PUR
21	14,982,497	15,013,906	*POTED*	LWK, CDX

Table S4. Genomic regions encompassing genes with a median fraction of overlap with class 4 ROA of > 0.9 in at last one population.

Genomic region			Genes	Populations
**Chr**	**Begin (bp)**	**End (bp)**		
1	12,998,504	13,002,353	*PRAMEF9*	TSI
1	206,317,458	206,332,104	*CTSE*	JPT, CHS
2	111,132,685	111,142,113	*LINC01106*	TSI
2	131,220,388	131,357,148	*POTEI*, ***CFC1***	TSI, CLM, PUR
4	9,212,382	9,370,796	*USP17L5, USP17L10, USP17L11, USP17L12, USP17L13, USP17L15, USP17L17, USP17L18, USP17L19, USP17L20, USP17L21, USP17L22, USP17L24, USP17252, USP17L26, USP17L27, USP17L28, USP17L29, USP17L30, USP17L6P, USP17L9P*	TSI
4	41,937,136	41,988,484	*TMEM33, DCAF4L1*	CDX
4	70,146,216	70,160,768	*UGT2B28*	ASW, LWK, YRI, ACB, ESN, GWD
5	69,140,495	74,867,509	*LOC653080, SERF1A, SERF1B,* ***SMN1****, SMN2, SMA4, GTF2H2B, SMA5, LOC441081, GUSBP9, NAIP, GTF2H2, LOC647859*	CDX
7	74,572,383	74,867,509	*NCF1C, CASTOR2*	CDX
8	7,118,140	7,143,880	*LINC00965*	PJL
8	7,812,534	7,946,611	*FAM66E, USP17L8, MIR548I3, FOXD4L5*	CDX, BEB, ITU, PJL
10	17,794,259	18,134,122	*TMEM236, MIR511*	CDX
10	48,155,942	48,158,691	*CTSLP2*	LWK, GWD
10	51,253,907	51,371,344	*PARGP1*	MXL, CDX, ITU
14	101,391,157	101,454,566	*SNORD113-1, SNORD113-2, SNORD113-4, SNORD113-5, SNORD113-6, SNORD113-7, SNORD113-9, SNORD114-1, SNORD114-2, SNORD114-3, SNORD114-4, SNORD114-5, SNORD114-6, SNORD114-7, SNORD114-8, SNORD114-9, SNORD114-10, SNORD114-11, SNORD114-12, SNORD114-13, SNORD114-14, SNORD114-15, SNORD114-16, SNORD114-17, SNORD114-18, SNORD114-19, SNORD114-20, SNORD114-21, SNORD114-22, SNORD114-23, SNORD114-24, SNORD114-25, SNORD114-26, SNORD114-27*	JPT, CDX
14	106,383,837	106,438,358	*FAM30A, ADAM6*	CEU, CHB, CIH, JPT, MXL, TSI, FIN, GBR, IBS, CLM, PUR, BEB, ESN, GWD, MSL
15	30,653,442	30,782,516	*CHRFAM7A, GOLGA8R, LOC100288203*	CDX
15	102,462,344	102,463,262	*OR4F4*	LWK, GWD
16	21,413,454	21,445,776	*NPIPB3, LOC100190986*	PUR
19	110,678	202,209	*OR4F17, LINC01002*	CDX
19	55,280,873	55,360,024	*ZNF818P, LOC101928804, KIR2DL1, KIR3DL1, KIR2DS4*	TSI, GBR, CDX, PEL, BEB, ITU, PJL STU
21	17,911,408	17,912,231	*MIR99A, MIRLET7C*	CDX

OMIM autosomal recessive genes are in **bold**.

Table S5. Genomic regions encompassing genes with a median fraction of overlap with class 3 ROA of > 0.9 in at last one population.

Genomic region			Genes	Populations
**Chr**	**Begin (bp)**	**End (bp)**		
1	248,756,130	248,790,429	*OR2T10, OR2T11*	ASW, LWK, YRI, ACB, GWD, MSL
2	114,256,660	114,258,727	*FOXD4L1*	CDX
4	70,146,216	70,160,768	*UGT2B28*	CEU, MXL, TSI, GBR, IBS, BEB, ITU, PJL, PEL
9	46,116,942	46,168,270	*LOC105376064, FAM27E2*	TSI
11	61,567,096	61,582,712	*FADS1, MIR1908*	ITU, STU
13	19,582,398	19,586,774	*LINC00442*	PEL
16	2,688,982	2,696,130	*FLJ42627*	CDX
16	21,413,454	21,445,776	*NPIPB3, LOC100190986*	CDX
19	43,715,942	43,752,798	*LOC284344*	KHV

Table S6. Genomic regions encompassing genes with a median fraction of overlap with class 2 ROA of > 0.9 in at last one population.

Genomic region			Genes	Populations
**Chr**	**Begin (bp)**	**End (bp)**		
8	41,284,173	41,284,246	*SNORD65B*	CHB
9	44,384,584	44,391,314	*LOC101927827*	MXL, BEB, ITU
11	62,334,482	62,334,543	*MIR6747*	CDX
15	25,440,067	25,440,148	*SNORD115-14*	CLM
20	62,159,775	62,168,723	*PTK6*	CHB

Table S7. Genomic regions encompassing genes with a median fraction of overlap with class 1 ROA of > 0.9 in at last one population.

Genomic region			Genes	Populations
**Chr**	**Begin (bp)**	**End (bp)**		
1	16,860,385	16,866,530	*LINC01783, FAM231B*	CDX
1	50,883,222	50,889,119	*DMRTA2*	JPT, CHS, CDX
1	120,839,004	120,855,681	*FAM72B*	MXL, CDX
1	142,697,420	142,713,605	*ANKRD20A12P*	CDX
2	91,824,708	91,970,153	*LOC654342, GGT8P*	MXL, CDX
3	48,658,274	52,029,958	*TMEM89, MIR6824, MIR4793, **SLC25A20**, ARIH2OS, P4HTM, WDR6, DALRD3, MIR435, **NDUFAF3**, MIR191, IMPDH2, QARS, MIR6890, **LAMB2**, LAMB2P1, CCDC71, KLHDC8B, C3orf84, C3orf62, MIR4271, **GPX1**, TCTA, APEH, UBA7, MIR5193, CAMKV, RBM5, **NPRL2**, CYB561D2, TMEM115, MIR4787, IQCF2, IQCF3, IQCF4, IQCF5, RRP9, PARP3, **ACY1**, RPL29*	CHB, GIH, JPT, MXL, TSI, GBR, CHS, PUR, CDX, PEL, KHV, BEB, ITU, PJL, STU
6	27,114,860	27,925,960	*HIST1H2AH, MIR3143, LOC100131289, HIST1H2BL, HIST1H2AI, HIST1H3H, HIST1H2AJ, HIST1H2BM, HIST1H4J, HIST1H4K, HIST1H2AK, HIST1H2BN, HIST1H2AL, HIST1H1B, HIST1H4L, HIST1H3J, HIST1H2AM, HIST1H2BO, OR2B2, OR2B6*	CEU, JPT, MXL, TSI, IBS, CHS, CDX, PEL
6	86,386,724	86,387,377	*SNHG5, SNORD50A, SNORD50B*	CDX
7	27,168,125	27,170,399	*HOXA4*	ITU
8	10,524,487	10,524,580	*MIR4286*	CHB
8	42,691,816	42,751,418	*THAP1, MIR4469*	TSI, JPT, FIN, IBS, ITU
9	43,608,372	43,630,730	*FAM74A7, SPATA31A6*	CEU, TSI, YRI, FIN, GBR, IBS, CDX, ACB, ITU, STU
9	44,384,584	44,391,314	*LOC101927827*	CDX
9	66,494,268	66,555,601	*PTGER4P2-CDK2AP2P2, LOC728673*	TSI, IBS, GWD, STU
9	99,671,356	99,704,572	*LOC441454, NUTM2G*	CEU, GBR, IBS, CLM, PUR
10	74,870,132	75,193,319	*NUDT13, SNORA11F, ECD, DNAJC9, **MRPS16**, MSS51*	MXL, TSI, FIN, GBR, IBS, PUR, PEL, STU
10	75,404,638	75,415,863	*SYNPO2L*	TSI
11	38,670,407	38,676,799	*LINC01493*	MXL, TSI
11	46,624,855	46,638,777	*HARBI1*	PEL
11	50,003,008	50,004,071	*OR4C12*	MXL, CDX
11	51,411,377	51,516,211	*OR4CA5, OR4C46*	MXL
11	55,110,676	55,433,572	*OR4A16, OR4C11, OR4P4, OR4S2, OR4C6*	ASW, CEU, GIH, LWK, MXL, TSI, YRI, GBR, CLM, PUR, CDX, ACB, BEB, GWD, ITU, MSL, PJL, STU
11	55,606,227	57,429,337	*OR5D16, OR5I1, OR10AG1, OR7E5P, OR5F1, OR5AS1, OR8I2, OR8H2, OR8H3, OR8J3, OR5J2, OR5T1, OR8K3, OR8K1, OR8U8, OR8U1, OR5AL1, OR5R1, OR5M8, OR5M11, OR5M10, OR5M1, TIMM10, MIR130A, CLP1*	MXL, CDX, PEL, KHV
11	89,819,117	89,820,299	*UBTFL1*	CDX
12	34,175,215	34,181,236	*ALG10*	JPT, CHS, CDX, KHV

12	111,374,405	111,395,622	*LINC01405, LOC10536980*	CDX, PEL
12	123,745,516	123,756,863	*CDK2AP1*	JPT
14	59,930,239	59,972,124	*GPR135, L3HYPDH, JKAMP*	CHB, CDX
14	67,908,571	67,908,647	*MIR5694*	CDX
15	24,686,273	24,693,114	*PWRN3*	ASW, CEU, JPT, LWK, TSI, FIN, IBS, CLM, PUR, ACB, BEB, GWD, ITU, MSL, PJL
15	42,491,767	42,491,864	*MIR627*	IBS
15	72,668,453	72,879,654	*TMEM202, MIR630*	GIH, CDX, BEB, PJL, STU
16	14,397,823	14,403,228	*MIR193B, MIR365A*	CDX
16	32,888,796	32,896,463	*SLC6A10P*	BEB
16	33,961,051	33,962,503	*LINC00273*	CEU, GIH, TSI, IBS, CLM, PUR, CDX, BEB, ITU, PJL, STU
16	34,403,801	34,404,762	*UBE2MP1, FRG2DP*	MXL, TSI, GWD, STU
16	46,723,557	46,920,386	*ORC6, SNORD148*	CDX, PEL, KHV
16	47,999,599	48,005,598	*LINC02134*	IBS, CDX, BEB
16	67,906,925	68,267,402	*EDC4, NRN1L, PSMB10, **LCAT**, DPEP3, ESRP2, MIR6773*	CHS, CDX, CHB
17	28,951,335	28,953,825	*SH3GL1P2*	TSI, GBR, IBS
19	28,281,400	28,284,848	*LINC00662*	CHB, CDX
20	25,593,572	26,188,914	*NANP, LINC01733, NCOR1P1, MIR663A*	CDX
20	29,637,583	30,619,984	*MLLT10P1, DEFB121, 1D1, MIR3193, **COX4I2**, ABALON, CCM2L*	CEU, CHB, GIH, MXL, IBS, CHS, CDX, PEL, KHV, BEB, ITU, PJL, STU
20	34,020,826	34,288,902	*GDF5OS, MIR1289-1, RBM12, ROMO1*	JPT, CDX, PEL, IBS, CLM
22	42,086,546	42,094,140	*C22orf46*	JPT
22	43,608,679	43,609,667	*LOC105373051*	CDX

OMIM autosomal dominant genes are underlined.
OMIM autosomal recessive genes are in **bold**.

Table S8. Genomic regions marginally enriched for autozygosity signals in the GIH, ITU, and PJL subgroups

Population			Genomic region				Number of windows	Minimum P_{perm}	RefSeq Genes & miRNA
ID	Name	Group	Chr	Begin (bp)	End (bp)	Length (bp)			
GIH	Gujarati	2	2	242,977,775	243,178,150	200,376	42	0.064	*LOC728323*
GIH	Gujarati	2	20	2,799,801	2,893,954	94,154	32	0.067	*PCED1A*, *VPS16*, *PTPRA*
ITU	Telugu	1	2	169,745,523	169,865,984	120,462	39	0.084	*G6PC2*, *ABCB11*
ITU	Telugu	1	6	137,323,081	137,441,791	118,711	3	0.083	*IL20RA*
ITU	Telugu	1	12	100,036,343	100,203,990	167,648	43	0.055	*ANKS1B*, *FAM71C*
ITU	Telugu	1	17	72,781,311	72,933,576	152,266	32	0.051	*TMEM104*, *GRIN2C*, *FDXR*, *FADS6*, *USH1G*, *OTOP2*, *OTOP3*
PJL	Punjabi	1	16	88,737,170	88,812,876	75,707	12	0.087	*SNAI3*, *RNF166*, *CTU2*, *MIR4722*, *PIEZO1*

Table S9. The 68 individuals removed during individual quality control check due to relatedness, likely erroneous sex assignment, or who did not cluster genetically with other members of their population.

Population		Individual ID	Reason for removal
**ID**	**Name**		
ASW	African American	NA19625	Intra-population AV relationship with NA20274
ASW	African American	NA19904	Intra-population PO relationship with NA19913
ASW	African American	NA20317	Intra-population PO relationship with NA20318
ASW	African American	NA20320	Intra-population PO relationship with NA20231
ASW	African American	NA20355	Intra-population PO relationship with NA20334
ASW	African American	NA20362	Intra-population PO relationship with NA20359
GIH	Gujarati	NA20900	Intra-population PO relationship with NA20882 & NA20891 (trio G001 in Pemberton *et al*. [207])
GIH	Gujarati	NA21109	Intra-population AV relationship with NA21135
LWK	Luhya	NA19042	Intra-population AV relationship with NA19042
LWK	Luhya	NA19334	Intra-population FS relationship with NA19331
ACB	Afro-Caribbean	HG02479	Intra-population FS relationship with HG02429
ITU	Telugu	HG03873	Inter-population FS relationship with HG03998
STU	Sri Lanka	HG03733	Intra-population FS relationship with HG03899
STU	Sri Lanka	HG03750	Intra-population PO relationship with HG03754
STU	Sri Lanka	HG03998	Inter-population FS relationship with HG03873
YRI	Yoruba	NA18870	Clusters with Esan (ESN) individuals
ESN	Esan	HG03294	Clusters with Yoruban (YRI) individuals
CDX	Dai	HG01798	Clusters with Han (CHB and CHS) and Kinh (KHV) individuals
CEU	European American	NA11932	Lies far from CEU cluster in comparisons with East Asian populations
CEU	European American	NA12383	Lies far from CEU cluster in comparisons with East Asian populations
CHB	Han Chinese (Beijing)	NA18550	Lies far from CHB cluster in comparisons with East Asian populations
CHB	Han Chinese (Beijing)	NA18628	Lies far from CHB cluster in comparisons with East Asian populations
JPT	Japanese	NA18976	Lies intermediate between JPT and the Han Chinese (CHB/CHS) cluster
MXL	Mexican Americans	NA19648	Lies far from MXL cluster and within the CLM/PUR clusters
MXL	Mexican Americans	NA19679	Lies far from MXL cluster and within the CLM/PUR clusters
GBR	Great Britain	HG00116	Lies far from GBR cluster in comparisons with European and East Asian populations
GBR	Great Britain	HG00120	Lies far from GBR cluster in comparisons with European and East Asian populations
CHS	Han Chinese (Southern)	HG00475	Lies far from CHS cluster in comparisons with East Asian populations

CHS	Han Chinese (Southern)	HG00500	Lies far from CHS cluster in comparisons with East Asian populations
CHS	Han Chinese (Southern)	HG00542	Lies far from CHS cluster in comparisons with East Asian populations
CLM	Colombian	HG01342	Lies far from CLM cluster in comparisons with European, East Asian, and Admixed populations
CLM	Colombian	HG01390	Lies far from CLM cluster in comparisons with European, East Asian, and Admixed populations
CLM	Colombian	HG01462	Lies far from CLM cluster in comparisons with European, East Asian, and Admixed populations
CLM	Colombian	HG01485	Lies far from CLM cluster in comparisons with European, East Asian, and Admixed populations
CLM	Colombian	HG01551	Lies far from CLM cluster in comparisons with European, East Asian, and Admixed populations
PUR	Puerto Rican	HG01108	Lies far from PUR cluster and within ASW/ACB clusters
PUR	Puerto Rican	HG01242	Lies far from PUR cluster and within ASW/ACB clusters
PUR	Puerto Rican	HG01063	Clusters with HG01108 and HG01242 and apart from the PUR cluster
PEL	Peruvian	HG02006	Lies far from PEL cluster in all comparisons
KHV	Kinh	HG02513	Lies very close to the CDX cluster
KHV	Kinh	HG02122	Lies intermediate between KHV and the Chinese (CHB/CHS/CDX) cluster
KHV	Kinh	HG02029	Lies intermediate between KHV and CHS clusters
KHV	Kinh	HG02032	Lies intermediate between KHV and CHS clusters
KHV	Kinh	HG02082	Lies intermediate between KHV and CHS clusters
ACB	Afro-Caribbean	HG02429	Lies far from ACB cluster in all comparisons except with MXL
BEB	Bengali	HG04161	Lies far from BEB cluster in comparisons with European and East Asian populations
BEB	Bengali	HG04162	Lies far from BEB cluster in comparisons with European and East Asian populations
ESN	Esan	HG03343	Lies far from ESN cluster in comparisons with African, European, and East Asian populations
ESN	Esan	HG03352	Lies far from ESN cluster in comparisons with African, European, and East Asian populations
ESN	Esan	HG03366	Lies far from ESN cluster in comparisons with African, European, and East Asian populations
GWD	Gambian	HG02610	Lies far from GWD cluster in comparisons with European and East Asian populations
GWD	Gambian	HG02624	Lies far from GWD cluster in comparisons with European and East Asian populations
GWD	Gambian	HG02642	Lies far from GWD cluster in comparisons with European and East Asian populations
GWD	Gambian	HG02666	Lies far from GWD cluster in comparisons with European and East Asian populations
MSL	Mende	HG03449	Lies far from MSL cluster in comparisons with African, European, and East Asian populations
MSL	Mende	HG03464	Lies far from MSL cluster in comparisons with African, European, and East Asian populations
MSL	Mende	HG03469	Lies far from MSL cluster in comparisons with African, European, and East Asian populations
MSL	Mende	HG03478	Lies far from MSL cluster in comparisons with African, European, and East Asian populations
MSL	Mende	HG03484	Lies far from MSL cluster in comparisons with African, European, and East Asian populations
STU	Sri Lankan	HG03898	Lies far from MSL cluster in comparisons with African, European, and East Asian populations

Additional File 2: Weighted Likelihood Inference of Autozygosity

STU	Sri Lankan	HG03955	Lies far from MSL cluster in comparisons with African, European, and East Asian populations
STU	Sri Lankan	HG03991	Lies far from MSL cluster in comparisons with African, European, and East Asian populations
LWK	Luhya	NA19332	Female with ~100% homozygosity on X chromosome
TSI	Toscani	NA20506	Female with ~100% homozygosity on X chromosome
TSI	Toscani	NA20530	Female with ~100% homozygosity on X chromosome
TSI	Toscani	NA20533	Female with ~100% homozygosity on X chromosome
FIN	Finnish	HG00361	Female with ~100% homozygosity on X chromosome
ESN	Esan	HG03511	Female with ~100% homozygosity on X chromosome
